# A single gene of a commensal microbe affects host susceptibility to enteric infection

**DOI:** 10.1038/ncomms11606

**Published:** 2016-05-13

**Authors:** Mi Young Yoon, Kyung Bae Min, Kang-Mu Lee, Yujin Yoon, Yaeseul Kim, Young Taek Oh, Keehoon Lee, Jongsik Chun, Byung-Yong Kim, Seok-Hwan Yoon, Insuk Lee, Chan Yeong Kim, Sang Sun Yoon

**Affiliations:** 1 Department of Microbiology and Immunology, Brain Korea 21 Project for Medical Sciences, Yonsei University College of Medicine, Seoul 03722, Korea; 2 ChunLab Inc., Seoul National University, Seoul 08826, Korea; 3 Department of Biotechnology, College of Life Science and Biotechnology, Yonsei University, Seoul 03722, Korea; 4 Institute for Immunology and Immunological Diseases, Yonsei University College of Medicine, Seoul 03722, Korea

## Abstract

Indigenous microbes inside the host intestine maintain a complex self-regulating community. The mechanisms by which gut microbes interact with intestinal pathogens remain largely unknown. Here we identify a commensal *Escherichia coli* strain whose expansion predisposes mice to infection by *Vibrio cholerae*, a human pathogen. We refer to this strain as ‘atypical’ *E. coli* (at*Ec*) because of its inability to ferment lactose. The at*Ec* strain is resistant to reactive oxygen species (ROS) and proliferates extensively in antibiotic-treated adult mice. *V. cholerae* infection is more severe in neonatal mice transplanted with at*Ec* compared with those transplanted with a typical *E. coli* strain. Intestinal ROS levels are decreased in at*Ec*-transplanted mice, favouring proliferation of ROS-sensitive *V. cholerae*. An at*Ec* mutant defective in ROS degradation fails to facilitate *V. cholerae* infection when transplanted, suggesting that host infection susceptibility can be regulated by a single gene product of one particular commensal species.

## Abstract (web summary)

The interactions between gut bacteria and enteric pathogens are poorly understood. Here, Yoon *et al*. show that subinhibitory antibiotic treatment in a mouse model leads to overgrowth of an *E. coli* strain carrying a catalase-encoding gene that enhances infection with the human pathogen *Vibrio cholerae*.

## Graphical abstract

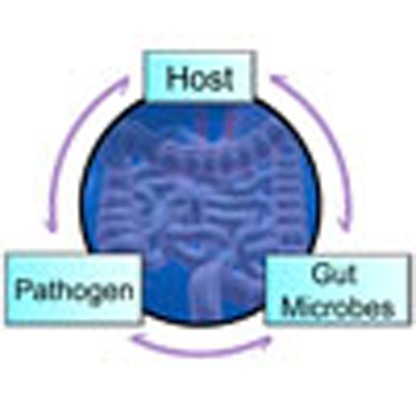

Commensal microbes, collectively termed gut microbiota, are considered to exist as a symbiotic community in the mucus layer lining the intestinal epithelium^1^,^2^. Due to the lack of means to isolate and preserve intestinal tissues, how these microbes maintain a dynamic and permanent co-evolutionary relationship with the host is not clearly understood. In some imaging studies, however, biofilm-like structures were successfully observed in animal intestines and surgically removed human appendices^3^,^4^. This suggests that (i) the resident microbes can act as a barrier against invading pathogens^5^ and (ii) enteric infections are the outcomes of multifaceted interactions between commensals, pathogens, and the host intestinal tissue.

Infectivity of pathogenic *E. coli* strains is controlled by the composition of commensal *E. coli* strains that can metabolize specific carbohydrates, thereby reducing their availability for consumption by pathogenic strains^6^. Anaerobic growth of both *E. coli* and *Salmonella enteria* serovar typhimurium can be supported by respiration using nitrate or tetrathionate, which are byproducts of the host inflammatory response^7^,^8^. Certain bacterial pathogens can increase their survival fitness inside the host intestine by catabolizing host-derived carbohydrates, the production of which is mediated by *Bacteroides thetaiotaomicron*, a distinct member of the gut microbiota^9^. The alteration of gut microbiota composition by antibiotic treatment has been shown to increase host susceptibility to intestinal infections by *S. enteria*^10^,^11^ and *Clostridium difficile*^12^. Furthermore, individuals for whom faecal transplantation resulted in restored microbiota have been shown to exhibit improved resistance to recurrent *C. difficile* infection^13^,^14^,^15^. However, due to the high degree of diversity of commensal microbes and the difficulty in culturing these strains, it has been difficult to correlate host susceptibility to infection with changes in the relative abundance of a defined subgroup of commensal microbes.

In this study, we seek to identify a commensal species and its genetic factor(s) that specifically influence host resistance to enteric infection. An *E. coli* strain with unusual features was significantly propagated during antibiotic treatment in mice and found to be responsible for modulating host susceptibility to infection by *V. cholerae* (*Vc*). We sequenced the whole genome of the *E. coli* strain and identified a novel catalase gene, disruption of which abrogated the infection-facilitating effects. This report provides novel insights into the role of gut microbiota in regulating the extent of intestinal infections.

## Results

### Antibiotic-treated mice became sensitive to *Vc* colonization

**Figure 1:**
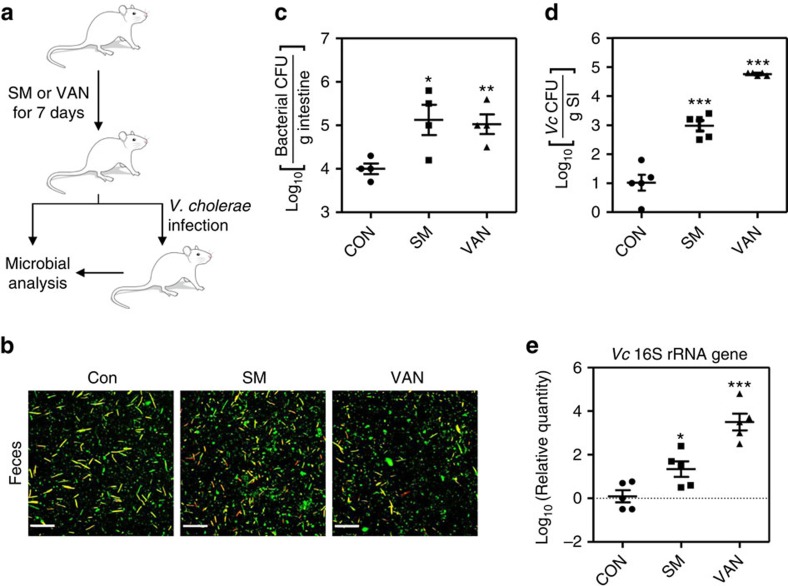
Antibiotic treatment induces changes in the gut microbiota composition and increases susceptibility to *Vc* infection. (**a**) Schematic diagram of the experimental procedure. Female CD-1 mice (5 to 6 weeks old) were treated with streptomycin (SM) and vancomycin (VAN) daily by oral gavage for 7 days. The daily doses administered were 1 mg for SM and 250 μg for VAN. At day 7 post-treatment, a subset of each treatment group was challenged with *Vc* for 2 days. (**b**) After antibiotic treatment, mouse faeces were collected and homogenized in PBS. The microbial cells in each suspension were visualized using a Live/Dead bacterial staining kit. Scale bar, 20 μm. (**c**) Mice (*n*=4) were killed and intestinal tissue lysates were prepared by homogenization. Bacterial cells that grew aerobically on plate count agar medium were enumerated and data are presented on a log scale. Values are displayed as means±s.e.m. for each treatment group. *P<0.05, **P<0.01 versus bacterial CFUs detected in the control group. (**d**) Antibiotic-treated mice (*n*=5) were infected with N16961 by oral gavage (∼10^7^ cells). At 2 days post-infection, mice were killed and the number of *Vc* cells recovered from the small intestine (SI) of each mouse was determined. Values are presented as means±s.e.m. and are displayed on a log scale. ***P<0.001 versus *Vc* CFUs detected in the control group. (**e**) Relative quantities of the *Vc* 16S rRNA gene in SI tissue homogenates (*n*=5) as determined by real-time PCR. Values were normalized to those of the *gapdh* gene. The ratios of the *Vc* 16S rRNA genes to the host *gapdh* gene are displayed on a log scale (means±s.e.m.). *P<0.05, ***P<0.001 versus the control group.

Commensal microbes play protective roles in host immunity against enteropathogenic infections^16^. To examine whether host susceptibility to intestinal infection is influenced by altered gut microbiota composition, we treated adult mice with low concentrations of streptomycin (SM, 1 mg per day) or vancomycin (VAN, 250 μg per day) daily for 7 days (Fig. 1a). Fluorescence staining of microbial cells recovered from mouse faeces revealed varying numbers of bacterial cells with differential shapes, with slightly more bacterial cells found in faeces obtained from SM-treated mice (Fig. 1b). Aerobic cultivation of mouse intestinal homogenates in plate count agar medium revealed that more bacterial colonies grew from the faeces recovered from SM-treated and VAN-treated mice compared with nontreated mice (Fig. 1c). These results suggest that our experimental conditions induced a change in the gut microbiota composition, rather than eliminating considerable portions of commensal microbes.

The adult mouse is not a natural host for *Vc*, the causative agent of the pandemic human disease cholera^17^. When control mice were infected with *Vc* via oral gavage, no significant *Vc* colonization was observed (Fig. 1d). However, a marked increase in *Vc* colonization was observed in antibiotic-treated mice; bacterial colonization was increased ∼100-fold and ∼10,000-fold in SM-treated and VAN-treated mice, respectively (Fig. 1d). We then measured the relative amount of the *Vc* 16S ribosomal RNA (rRNA) gene in the small intestine of each group. Consistent with the increased *Vc* colonization, a higher level of the *Vc* gene was detected in antibiotic-treated mice and this increase was most significant in VAN-treated mice (Fig. 1e). Altogether, these results demonstrate that administration of low concentrations of antibiotics alters the mouse gut microbiota composition, which subsequently results in increased host vulnerability to *Vc* colonization.

### An at*Ec* strain proliferated on antibiotic treatment

We then quantitated the relative abundance of different commensal microbe phylogenetic groups in each treatment group by real-time PCR using group-specific primer sets (Supplementary Table 1). Genomic DNA extracted from small intestine tissue lysates was subjected to amplification and normalized to the level of the host *gapdh* gene. The relative quantity of the γ-proteobacteria 16S rRNA gene was markedly increased in VAN-treated mice (Supplementary Fig. 1a). Subsequent family-level and species-level examinations indicated that the levels of the Enterobacteriaceae and *E. coli* 16S rRNA genes were remarkably increased in VAN-treated mice and were also increased, albeit to a lesser extent, in the SM-treated group (Supplementary Fig. 1b,c). In contrast, the relative quantities of the marker genes for selective detection of *Bifidobacterium*, *Lactobacillus* and *Bacteroides* were similar between all groups (Supplementary Fig. 1d–f). These results demonstrate that (i) the abundance of *E. coli* was specifically increased during treatment with SM or VAN, and (ii) this increase likely accounted for the elevated levels of Enterobacteriaceae and γ-proteobacteria class bacteria in the antibiotic-treated groups.

**Figure 2:**
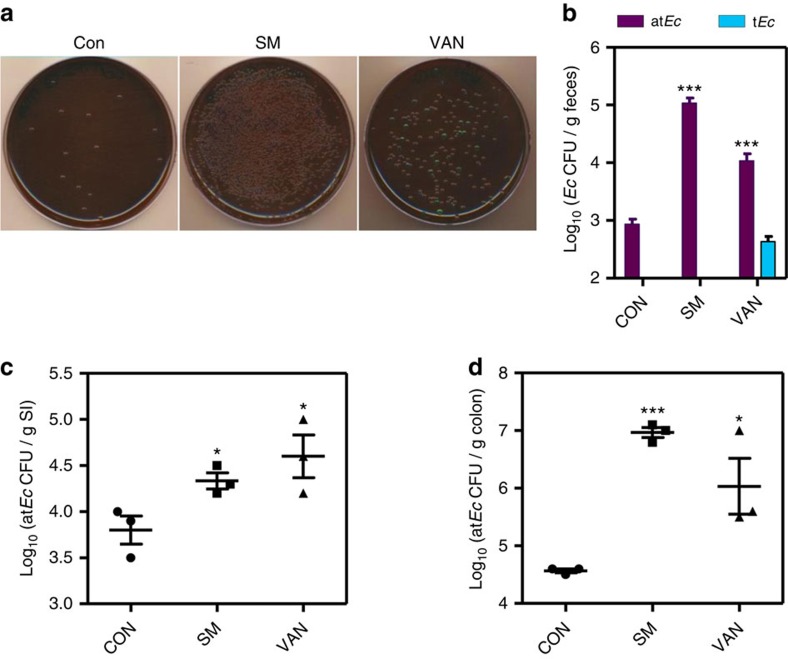
Atypical *E. coli* cells proliferate rapidly in response to antibiotic treatment. (**a**) Representative images of EMB plates inoculated with aliquots of mouse faecal suspensions. Mouse faeces were collected and homogenized in PBS before inoculation. (**b**) Quantification of at*Ec* and t*Ec* strains recovered from mouse faeces collected from each treatment group. The CFUs of at*Ec* and t*Ec* cells are shown in purple and blue bars, respectively. Values are expressed as means±s.e.m. and are displayed on a log scale. ***P<0.001 versus at*Ec* CFUs detected in the control group. (**c**) Quantification of at*Ec* colonies grown from the small intestine homogenates of each group. Values are expressed as means±s.e.m. and are displayed on a log scale. *P<0.05 versus the control group. (**d**) Quantification of at*Ec* colonies grown from the mouse colon homogenates of each group. Values are expressed as means±s.e.m. and are displayed on a log scale. ***P<0.001, *P<0.05 versus the control group.

Next, suspensions of mouse faeces were cultivated on eosin methylene blue (EMB) agar, a selective medium for Gram-negative bacteria, especially those belonging to the Enterobacteriaceae family^18^. Consistent with our DNA-based assay results, more colonies grew from faeces obtained from SM-treated and VAN-treated mice (Fig. 2a). Species identification was performed on several colonies and revealed that all were *E. coli.* This finding further supports the results shown in Supplementary Fig. 1. Intriguingly, however, a majority of the bacterial cells grew as colourless colonies on the EMB plates (Fig. 2a). Typical *E. coli* strains can ferment lactose, thereby yielding colonies with a distinctive metallic green sheen when grown on EMB plates^19^. As shown in Fig. 2b, the numbers of atypical (that is, colourless) *E. coli* (termed at*Ec*) colonies increased to ∼10^5^ and ∼2.5 × 10^4^ per g of faeces in SM-treated and VAN-treated mice, respectively. Notably, typical green colonies (termed t*Ec*) were also recovered in the VAN-treated group (Fig. 2b). We then performed an RAPD assay to examine the extent of genomic diversity among these *E. coli* isolates. Sixteen strains (14 colourless, 1 green and 1 intermediate green) were included in this analysis. Although the amplification products were almost identical among the at*Ec* strains, these products were distinct from those obtained from the typical green and intermediate green strains (Supplementary Fig. 2). This result suggests that (i) at*Ec* strains possess a distinct genome, and (ii) the antibiotic-induced propagation of at*Ec* strains was likely mediated by clonal expansion. The numbers of at*Ec* strains were also significantly increased in the small intestine and the colon of SM-treated and VAN-treated mice (Fig. 2c,d). In each group, at*Ec* strains were more prevalent in the colon than in the small intestine, indicating that the colon is a more suitable habitat for this strain.

We next asked whether propagation of the at*Ec* strain was invariably observed in mice that received a different antibiotic regimen (Supplementary Fig. 3a). Treatment of adult mice for 4 weeks with a mixture of four different antibiotics in the drinking water revealed a similar increase in the number of colourless colonies, which were later identified as *E. coli*, on EMB plates (Supplementary Fig. 3b). Of particular note, the mean mouse colon weight was increased in the antibiotic-treated group (∼0.2 g) versus the control group (∼0.16 g), an apparent host phenotypic change in response to antibiotic treatment (Supplementary Fig. 3c). On subsequent *Vc* infection, antibiotic-treated mice exhibited significantly increased fluid accumulation (FA) in their small intestines (Supplementary Fig. 3d). The extent of FA has been shown to depend on the degree of *Vc* infectivity of the host^20^. Most importantly, ∼50-fold more *Vc* cells were recovered from the intestines of antibiotic-treated mice (Supplementary Fig. 3e). Altogether, these findings further suggest that the at*Ec* strain is highly proliferative under antibiotic stress and that *Vc* infection is facilitated by the increased abundance of the at*Ec* strain.

### The at*Ec* strain possesses an extra catalase gene

We next sought to understand the molecular basis underlying the positive effect of the at*Ec* strain on *Vc* colonization. To this end, we sequenced the genomes of the at*Ec* and t*Ec* strains and compared them with the *E. coli* K12 genome. The at*Ec* strain was found to have the largest genome (5.24 Mbp), whereas the t*Ec* and K12 genomes were 4.72 Mbp and 4.64 Mbp, respectively (Supplementary Fig. 4a). Alignment of the at*Ec* and t*Ec* genomes using the Maximal Unique Matcher algorithm^21^ revealed that most of the regions overlapped with each other (Supplementary Fig. 4b, red diagonal line). However, many similar genetic elements were found to be highly scattered over the entire genomes, revealing a high degree of dissimilarity between the two genomes (Supplementary Fig. 4b). Of particular note, the 3′ region of the *lacY* gene (which encodes lactose permease) was deleted in the at*Ec* strain, whereas the full-length *lacY* gene was detected in the t*Ec* and K12 strains (Supplementary Fig. 4c). The presence of a defective *lacY* gene explains why the at*Ec* strain formed colourless colonies on the lactose-containing EMB plates.

We then carried out genome-based clustering analysis using two isolates and representative *E. coli*/*Shigella* strains. In a dendrogram constructed based on the average nucleotide identity (ANI), the t*Ec* and at*Ec* strains were quite distant from *E. coli* K12 (Supplementary Fig. 5). Futhermore, t*Ec* and at*Ec* were relatively distant from each other, with ANI of 99.1%, indicating that the two strains belong to different phylogenetic lineages within the *E. coli* group (Supplementary Fig. 5).

Importantly, the comparison with the t*Ec* genome showed that the at*Ec* genome contains an extra gene encoding a catalase, a critical enzyme that protects cells from ROS-mediated oxidative damage (Supplementary Fig. 6a). Activity-based catalase assays indicated that the at*Ec* strain produced three distinct catalases, including two that were also detected in the t*Ec* strain (Supplementary Fig. 6b, black arrowheads). The extra catalase (termed eKatE) expressed in the at*Ec* strain was only ∼61% identical to KatE on the basis of amino-acid sequence (Supplementary Fig. 6c). Among 4,045 *Escherichia* and *Shigella* genomes available in public databases, only one strain, *E. coli* K02, has a gene of identical sequence to that of the at*Ec* strain. Outside the *Escherichia*/*Shigella* group, an *eKatE*-like gene (with >90% nucleotide sequence identity) was found only in *Serratia* (γ-proteobacteria) and *Frankia* (Actinobacteria) species (Supplementary Fig. 6d). This information suggests that the *eKatE* gene might have been horizontally transferred from another species with a different degree of relatedness.

To provide supporting evidence for horizontal transfer of the *eKatE* gene, we performed several bioinformatics analyses. First, we constructed two different phylogenetic trees based on amino-acid sequences of KatG and KatE proteins, respectively (Supplementary Fig. 7). In the KatG-based tree, KatG proteins from t*Ec* and at*Ec* strains were clustered together with those of other *E. coli* strains (Supplementary Fig. 7a, black arrows). Likewise, evolutionary distance was not detected between KatE proteins from both strains (Supplementary Fig. 7b, black arrows). In contrast, *eKatE*-encoded catalase was distinctly clustered with proteins from unrelated species (for example, of the genera *Serratia*, *Frankia*, *Lonsdalea* and *Rouxiella*; Supplementary Fig. 7b, red arrow). Second, tetranucleotide frequency was analysed for the *katE* genes found in 33 representative *E. coli* strains. The tetranucleotide frequency of the *eKatE* gene exhibited the lowest correlation coefficient when compared with other values in the matrix table (Supplementary Table 2). In contrast, *katE* genes of the at*Ec* or t*Ec* strain were similar to other *katE* genes of *E. coli* origin in terms of the tetranucleotide frequency. Third, we also measured codon adaptation index (CAI) of three catalase genes (*katG*, *katE* and *eKatE*) of the at*Ec* strain. The CAI of the *eKatE* gene clearly deviated from that of the other two genes (Supplementary Table 3). Moreover, GC content of the *eKatE* gene was significantly lower than that of *katG* and *katE* genes. Fourth, shared synteny was observed for genes encoding transposase in at*Ec* and *E. coli* K02 strains (Supplementary Fig. 8). Genes encoding transposases are also detected in *Serratia* and *Frankia* species (Supplementary Fig. 8). More importantly, *ccdBA* genes are present near the *eKatE* gene in the at*Ec* chromosome (Supplementary Fig. 9). These two genes are known to be plasmid-borne and produce the CcdA/CcdB toxin-antitoxin module that is involved in plasmid maintenance in *E. coli*^22^. Altogether, these analyses strongly suggest that the at*Ec* strain acquired the *eKatE* gene by horizontal gene transfer from an outside mobile genetic source.

### The at*Ec* strain is highly resistant to H_2_O_2_

**Figure 3:**
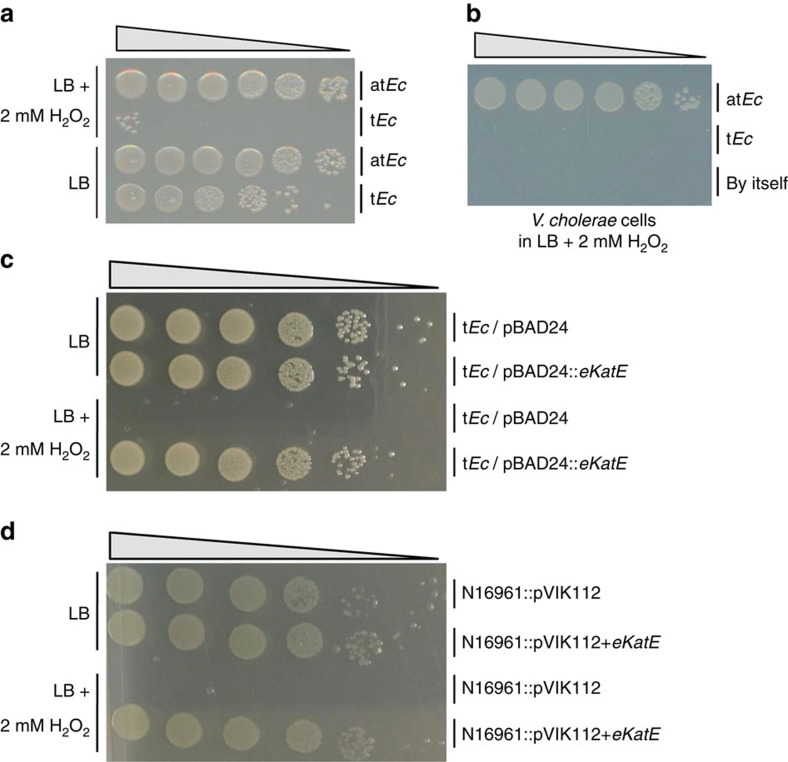
The at*Ec* strain is resistant to H_2_O_2_. (**a**) Viable cell numbers of at*Ec* and t*Ec* strains after growth in LB for 3 h in the presence (top two rows) or absence (bottom two rows) of 2 mM H_2_O_2_. Serial dilutions of bacterial cultures were spot-inoculated onto LB plates. (**b**) Viable cell numbers of *Vc* N16961. Overnight cultures of N16961 cells were diluted 100-fold in LB+2 mM H_2_O_2_ that had been precultured for 2 h with at*Ec* or t*Ec*. N16961 was grown for a further 4 h. The *Vc* CFUs in each culture were determined by growing serial dilutions on LB+200 μg ml^−1^ SM plates. (**c**) Viable cell numbers of t*Ec* strains harbouring pBAD24 or pBAD24::*eKatE* after growth in LB for 3 h in the absence (top two rows) or presence (bottom two rows) of 2 mM H_2_O_2_. Serial dilutions of bacterial cultures were spot-inoculated onto LB plates. (**d**) Viable cell numbers of *Vc* N16961 strains with chromosomally integrated pVIK112 or pVIK112+*eKatE* plasmid after growth in LB for 3 hrs in the absence (top two rows) or presence (bottom two rows) of 2 mM H_2_O_2_. Serial dilutions of bacterial cultures were spot-inoculated onto LB+200 μg ml^−1^ SM plates.

On the basis of our native gel-based activity assay (Supplementary Fig. 6b), *eKatE*-encoded catalase appeared to have a stronger enzymatic activity than KatG or KatE, suggesting that the at*Ec* strain might be more resistant to H_2_O_2_ than the t*Ec* strain. In LB supplemented with 2 mM H_2_O_2_, at*Ec* cells grew completely normally, as they did in plain LB (Fig. 3a). In contrast, growth of the t*Ec* strain was significantly inhibited in the presence of H_2_O_2_ (Fig. 3a), indicating that the production of eKatE may render the at*Ec* strain resistant to H_2_O_2_. *Vc* cells grew normally in LB+2 mM H_2_O_2_ that had been preinoculated with at*Ec* for 2 h, whereas no growth was observed in the same medium that had been pretreated with t*Ec* for the same period of time (Fig. 3b). These results suggest that at*Ec* cells can detoxify H_2_O_2_, thereby helping *Vc* cells proliferate in the presence of ROS stress. An additional 18 strains that formed colourless colonies on the EMB plates also produced significant levels of eKatE, further supporting the idea that the at*Ec* strain had clonally expanded during antibiotic treatment (Supplementary Fig. 10). The relative quantity of the *eKatE* gene in small intestine tissue lysates was increased in the SM-treated group and to an even greater extent in the VAN-treated group (Supplementary Fig. 11). The increase in the level of the *eKatE* gene was proportional to that of the *E. coli* 16S rRNA gene (Supplementary Fig. 1c). This result further indicates that the increased population of *E. coli* in antibiotic-treated mice can be attributed to the proliferation of at*Ec* strains.

The t*Ec* strain also produces KatG and KatE, two distinct catalases (Supplementary Fig. 6b). To further verify the role of eKatE in H_2_O_2_ resistance, we constructed recombinant t*Ec* and *Vc* strains that express a plasmid-borne or a chromosomally inserted *eKatE* gene, respectively. A DNA element encompassing both the *eKatE* gene open reading frame and its endogenous promoter was used for cloning. The recombinant t*Ec* strain harbouring the pBAD24::*eKatE* plasmid was found to be resistant to 2 mM H_2_O_2_ and grew completely normally in the presence of 2 mM H_2_O_2_, as in plain LB media (Fig. 3c). Moreover, a *Vc* N16961 strain expressing the *eKatE* gene was equally resistant to 2 mM H_2_O_2_ (Fig. 3d). These results demonstrate that the *eKatE*-encoded catalase is responsible for the ROS resistance detected in at*Ec* cells. Besides the unique presence of *eKatE* gene in the at*Ec* strain, genetic repertoires for oxidative stress responses are almost identical between the two strains. In each strain, 57 genes were identified that are known or presumed to be involved in oxidative stress response. Among these, 56 genes are found in both genomes, except for at*Ec*_0417 (*eKatE*) and t*Ec*_2780 genes (Supplementary Table 4).

### *Vc* infection is severe in the at*Ec*-transplanted infant mice

**Figure 4:**
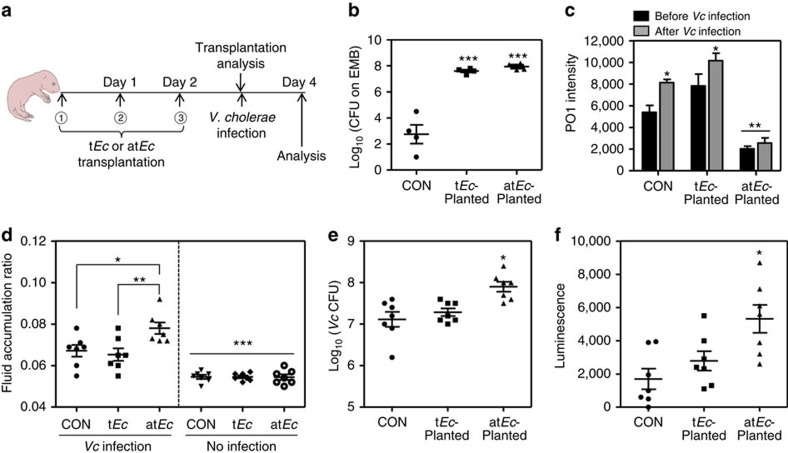
Infant mice transplanted with at*Ec* exhibit increased susceptibility to *Vc* infection. (**a**) Schematic diagram of the experimental procedure. Five-day-old infant mice were orogastrically transplanted three times with either the at*Ec* or t*Ec* strain (10^7^ cells). On day 3 post-transplant, mice were infected with 10^6^ N16961 cells. (**b**) To assess the transplantation efficiency, a subset of the mice in each group (*n*=4) were killed and intestinal tissue homogenates were obtained. These homogenates were inoculated into EMB medium to determine the number of ingested bacterial cells. Values are expressed as means±s.e.m. and are displayed on a log scale. ***P<0.001 versus the number of bacterial colonies from the control group. (**c**) Mouse intestinal tissue lysates obtained from each group (*n*=4) were stained with 100 μM PO1 for 30 min to detect ROS. Values are expressed as means±s.e.m. and are displayed in each bar. *P<0.05 versus PO1 signals from the ‘before *Vc* infection’ groups. **P<0.01 versus the signals of all the other groups. (**d**) Each group of mice was challenged with *Vc* (*n*=7, left panel) or left uninfected (*n*=7, right panel). The fluid accumulation (FA) ratio of each group was measured and plotted on a linear scale. *P<0.05 versus the FA ratio of the *Vc*-infected control group. **P<0.01 versus the FA ratio of the *Vc*-infected t*Ec*-transplanted mice. *** indicates the FA ratios of the noninfected groups were significantly different from those of all *Vc*-infected groups (*P*<0.005). (**e**) The number of *Vc* cells that colonized the mouse intestine (*n*=7 in each group) was determined by CFU counting. Values are expressed as means±s.e.m. and are displayed on a log scale. *P<0.05 versus *Vc* CFUs of the control or t*Ec*-transplanted group. (**f**) Infant mice (*n*=7 per group) were infected with a *Vc ctxAB* promoter fusion reporter strain. The level of bioluminescence was measured in each mouse intestinal tissue homogenate. Values are expressed as means±s.e.m. and are displayed on a linear scale. *P<0.05 versus the bioluminescence level in control mice.

Infant mice have been widely used as a surrogate host to study *Vc* infection *in vivo*^23^,^24^. We found that antibiotic-treated mice exhibited a substantially altered gut microbiota composition and increased susceptibility to *Vc* colonization. To define causality in the relationship between the increased intestinal population of at*Ec* and increased host sensitivity to *Vc* infection, infant mice were challenged with *Vc* following daily transplantation with 10^7^ at*Ec* or t*Ec* cells for 3 days (Fig. 4a). Transplantation of t*Ec* or at*Ec* was efficiently achieved, as demonstrated by the recovery of ∼4 × 10^7^ t*Ec* and ∼9 × 10^7^ at*Ec* cells from mouse intestines at 3 days post transplantation (Fig. 4b). Significantly lower bacterial loads were detected in the control group, indicating that bacterial species belonging to the Enterobacteriaceae family represent only a minor proportion of the total commensal microbes in the infant mouse (Fig. 4b). Importantly, when mouse intestinal tissue homogenates were stained with PO1, a ROS-sensitive fluorescent dye^25^, a marked decrease in the fluorescence signal was observed in the at*Ec*-transplanted group but not in its t*Ec*-transplanted counterpart (Fig. 4c). The PO1-specific signal was somewhat increased in the intestines of t*Ec*-transplanted mice compared with those from the control group (Fig. 4c). This finding indicates that at*Ec* cells can also readily degrade host-derived ROS *in vivo*. The PO1-specific signals were increased in control and t*Ec*-transplanted groups after *Vc* infection (Fig. 4c), suggesting that *Vc* infection stimulates ROS production in the mouse intestine. Of particular importance, such an increase was not observed in the at*Ec*-transplated group (Fig. 4c), further suggesting that an abundant population of at*Ec* cells can control intestinal ROS levels in the host.

Subsequent *Vc* infection resulted in a noticeable increase in the level of intestinal FA in at*Ec*-transplanted mice (Fig. 4d), whereas the FA ratios of the t*Ec*-transplanted and control groups were comparable. Significantly active *Vc* colonization and high levels of the *ctxAB* promoter were also detected in mice transplanted with at*Ec* (Fig. 4e,f). These results demonstrate that high loads of at*Ec* cells inside the host intestine generate conditions that facilitate *Vc* infection.

### Increased *Vc* infection is due to *eKatE*-encoded catalase

**Figure 5:**
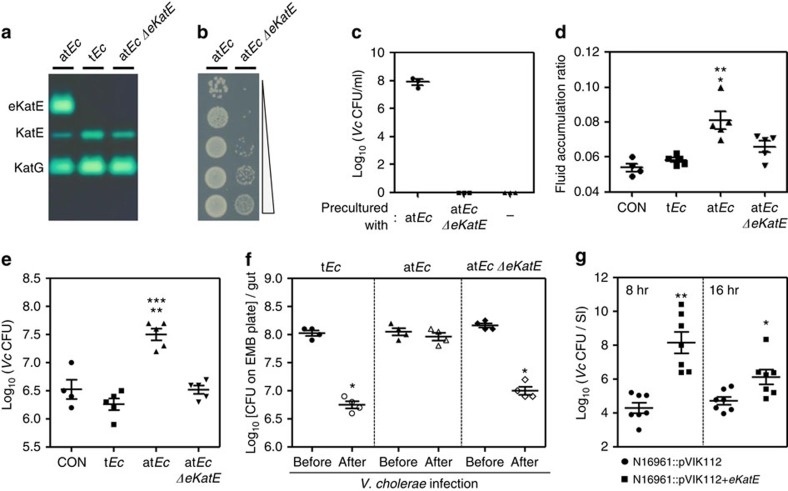
The *eKatE*-encoded catalase plays a critical role in at*Ec*-mediated enhancement of *Vc* infectivity. (**a**) Construction of an at*Ec eKatE* deletion mutant. Bacterial extracts were loaded on a 7.5% nondenaturing polyacrylamide gel, electrophoresed to allow protein separation and then stained for catalase activity. (**b**) Serial dilutions of bacterial cultures (at*Ec* strain and its Δ*eKatE* mutant) were inoculated onto LB plates after growth in LB+2 mM H_2_O_2_ for 3 h. (**c**) An overnight culture of N16961 was diluted 100-fold into LB+2 mM H_2_O_2_ that had been precultured for 2 h with at*Ec* or its Δ*eKatE* counterpart. N16961 cells were grown for a further 4 h. *Vc* viability was determined by CFU counting. (**d**) *Vc*-induced FA ratios in groups of infant mice (*n*=5 per group) that had been transplanted with t*Ec*, at*Ec* or at*Ec* Δ*eKatE* cells. Non-transplanted mice (*n*=4) were used for the control infection. **P<0.005 versus the FA ratio of the control or t*Ec*-transplanted group. *P<0.05 versus the FA ratio of Δ*eKatE*-transplanted mice. (**e**) The numbers of *Vc* N16961 cells recovered from the intestines of each group are expressed as means±s.e.m. and are displayed on a log scale. **P<0.005 versus *Vc* CFUs from the control group. ***P<0.001 versus *Vc* CFUs from t*Ec*-transplanted or Δ*eKatE*-transplanted mice. (**f**) The number of each *E. coli* strain recovered from infant mice (*n*=4 per group) before (solid) and after (open) *Vc* infection was determined; values are expressed as means±s.e.m. and are displayed on a log scale. *P<0.01 versus CFUs from the ‘before’ group. (**g**) Adult mice (*n*=14 per group) were infected with either *Vc* N16961::pVIK112 (circles) or N16961::pVIK112+*eKatE* (squares) by oral gavage. At 8 and 16 h post-infection, seven mice in each infection group were killed and bacterial numbers present in small intestine were determined. Values are expressed as means±s.e.m. **P<0.001, *P<0.05 versus control infection. Bacterial suspensions were prepared from mid-log phase cultures at ∼10^14^ per ml and 200 μl of suspensions were used for oral gavage.

We then examined whether the catalase activity of the *eKatE* gene product plays a role in at*Ec*-mediated enhancement of *Vc* infection. To address this, we constructed an *eKatE* in-frame deletion mutant of at*Ec* strain. The data shown in Fig. 5a clearly demonstrate the lack of *eKatE*-encoded catalase activity of the mutant. Disruption of the *eKatE* gene abrogated the H_2_O_2_ resistance of the at*Ec* strain (Fig. 5b). The at*Ec* Δ*eKatE* mutant failed to protect *Vc* cells against H_2_O_2_ stress in our *in vitro* co-culture system (Fig. 5c). Importantly, the extent of *Vc* infection-mediated FA induction was significantly reduced in Δ*eKatE*-transplanted infant mice compared with at*Ec*-transplanted infant mice (Fig. 5d). Moreover, the facilitatory effect of at*Ec* transplantation on *Vc* colonization disappeared when the *eKatE* gene was deleted. As shown in Fig. 5e, 10-fold higher numbers of *Vc* cells were recovered after 24-h infection in at*Ec*-transplanted infant mice, compared with t*Ec*- or Δ*eKatE*-transplanted groups. It is of particular interest that at*Ec* remained colonized during the 24-h *Vc* infection period, whereas the abundance of t*Ec* and the Δ*eKatE* mutant substantially decreased in response to *Vc* infection (Fig. 5f). These findings suggest that (i) the at*Ec* strain possesses better ‘colonization fitness’ under *Vc* infection-induced host stress conditions and (ii) *Vc in vivo* colonization likely occurs as a consequence of co-operative interaction with at*Ec* cells in a catalase-dependent manner.

Finally, we examined whether the H_2_O_2_-resistant *Vc* strain shown in Fig. 3d exhibited a superior colonization capability in conventional adult mice. The N16961::pVIK112+*eKatE* strain colonized significantly better than the control strain; at 8 h post-infection ∼10,000-fold more bacterial cells remained colonized in the mouse small intestine (Fig. 5g). Although lower numbers of bacterial cells were recovered after 16 h of infection, the *eKatE*-expressing cells still colonized ∼13.8-fold better than the control cells (Fig. 5g). This result demonstrates that *Vc* colonization also occurs more readily when *Vc* alone can handle ROS stress.

## Discussion

The gut microbiota and products encoded by its genome (that is, the gut microbiome) play critical roles in human health. Commensal bacteria that reside in the host intestine contribute to the development of a functional intestinal immune system^26^. The gut microbiome, which is considered to supplement the human genome with >100-fold more genes, contains genes that produce digestive enzymes lacking in humans^27^. Furthermore, phylum-level changes in their composition account for the differential propensity of individuals to develop obesity^28^. Along with these important functions, commensal microbes also participate in regulating host defenses against the invasion of pathogenic bacteria^16^,^29^. In this study, we isolated a commensal *E. coli* strain that exhibits exceptional resistance to ROS and demonstrated that such an atypical *E. coli* strain, when abundantly present in the host intestine, can enhance host susceptibility to enteric infection.

Our results suggested that the at*Ec* strain was slightly more resistant to SM than the t*Ec* strain; in the presence of SM (32 μg ml^−1^), at*Ec* cells grew slightly better than t*Ec* cells (red arrow, Supplementary Fig. 12). However, both at*Ec* and t*Ec* strains were equally sensitive to ampicillin and tetracycline (data not shown). In addition, both strains showed no sensitivity to VAN, an effective antibiotic against Gram-positive organisms (data not shown). On the basis of these results, it appears that the at*Ec* strain is not more resistant to antibiotics and therefore the abundant population of at*Ec* in antibiotic-treated mice is not caused by its superior capability to survive antibiotic treatment.

On the other hand, host-mediated changes may play a critical role in creating an environment that stimulates the propagation of at*Ec* cells in antibiotic-treated mice. Accumulating evidence suggests that an increase in LPS inside the host intestine stimulates ROS production^30^,^31^,^32^. During antibiotic treatment, the leve of Enterobacteriaceae, a large family of Gram-negative bacteria, was greatly increased. Therefore, it is likely that the amount of LPS released from those Gram-negative species may also be significantly increased. Consistent with this notion, the data in Fig. 4c show that the PO1 signal was noticeably increased in t*Ec*-transplanted mice, indicating that the increased population of t*Ec* cells inside the mouse intestine resulted in elevated ROS levels. Stable transplantation of the at*Ec* strain, however, resulted in a significant decrease in the level of ROS inside the host intestine (Fig. 4c). This change in the intestinal environment presumably helps *Vc*, which is known to be ROS-sensitive^33^, colonize and exert pathogenic effects. Recently, Lupp *et al*.^34^ reported that the relative abundance of nonpathogenic *E. coli* was increased during chemically induced intestinal inflammation, a process that generates excessive ROS. However, no basis was provided for such a population change. It will be very interesting to see whether the *E. coli* strain described in their study shares common phenotypes with the at*Ec* strain that we identified in the current study. The robust activity of the *eKatE*-encoded catalase helped the at*Ec* strain overcome surrounding environmental changes, which likely involved the accumulation of high levels of ROS. Although it remains unclear how much ROS was actually produced during antibiotic treatment in our model system, the at*Ec* strain must have competitive survival fitness under conditions of host-mediated ROS production.

*Vc* colonization and cholera toxin-induced intestinal fluid accumulation were increased in at*Ec*-transplanted infant mice, but not in mice transplanted with at*Ec* cells lacking the *eKatE* gene. These results indicated that the pathogenesis of *Vc* infection was critically influenced by gut microbiota composition and a microbiome gene product that regulates intestinal ROS level *in vivo*. Diverse virulence determinants have been reported to play distinct roles in *Vc* pathogenesis in different animal models. For example, persistent colonization of *Vc* in adult mice depends on accessory toxins (that is, hemolysin and RTX toxin), but not on toxin co-regulated pilus^35^. Toxin co-regulated pilus, however, is essential for bacterial colonization in infant mice and infant rabbits^36^,^37^. In addition, cholera toxin promotes bacterial colonization in adult rabbits^38^, but not in infant rabbits^36^. These previous results strongly suggest that host age is an important factor that affects susceptibility to *Vc* infection. Since changes in microbiota composition are clearly observed with aging^39^,^40^,^41^, how *Vc* infectivity is modulated by age-dependent changes in microbiota composition must be further addressed.

The development of cholera vaccines has been hampered, in part, by the difficulty in assessing the efficacy of a candidate vaccine economically using adult mice. Our results suggest that adult mice with an increased population of at*Ec* cells can develop cholera-like symptoms. The activity of catalase encoded by the *eKatE* gene appears to be significantly higher than that of other well-characterized catalases^42^. We showed that a recombinant *Vc* strain that produces *eKatE*-encoded catalase was fully resistant to 2 mM H_2_O_2_ and this ROS-resistant *Vc* strain exhibited enhanced colonization capability in a normal adult mouse. Provided that virulence is unaffected by *eKatE* gene expression, this recombinant *Vc* strain may have potential application as a challenge strain in future vaccine development.

As shown in Supplementary Data 1, 269 different *katE*-encoded catalases were identified in the human gut microbiome catalogue. This indicates that at least 269 bacterial species can produce proteins identical or similar to the KatE catalase in human intestine. Of note, KatE proteins produced by the species belonging to the Proteobacteria phylum exhibit the highest sequence similarities with that of the at*Ec* strain. The genomes of 50 bacterial species, mostly in the same phylum, encode KatG catalases. Meanwhile, in the mouse gut microbiome catalogue, 93 and seven *katE*- and *katG*-encoded catalases were identified, respectively. Interestingly, Proteobacteria is not a major group that produces KatE catalase in mouse intestine. Altogether, these results suggest that (i) KatE might be produced in larger quantities than KatG in both human and mouse intestines, and (ii) different bacterial species contribute to the production of KatE catalases in human versus mouse intestine. Currently, detailed information is lacking in regards to the relative abundances of individual catalase producers in the gut microbiota, and more information is needed to more definitively compare overall catalase activity in human versus mouse intestines. We expect that such information would prove useful in identifying the mechanisms of human-restricted tropism of bacterial infections, including that by *V. cholerae*.

**Figure 6:**
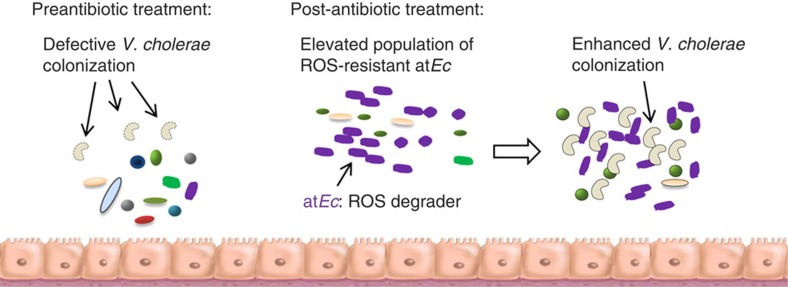
Summary of antibiotic-induced proliferation of at*Ec* and its impact on host susceptibility to *Vc* infection. Phylogenetically diverse bacterial cells that were originally present in the untreated host are depicted in different colours and shapes. Defective and enhanced colonization of *Vc* cells are denoted with dotted and solid lines (comma-shaped), respectively. The increased population density of at*Ec* is indicated by the increased number of purple cells.

The commensal species that modulate host susceptibility to disease are beginning to be defined^9^,^43^. However, the underlying genetic determinants that modulate this susceptibility have not yet been identified. The ability of at*Ec* to facilitate *Vc* infection was completely abrogated by the deletion of a single gene, *eKatE*. This finding demonstrates that the level of ROS inside the host intestine, which must be carefully regulated for protection against pathogenic invaders, can be controlled by a single-microbiome gene product. We anticipate that our results (summarized in Fig. 6) will stimulate the assembly of a database of microbiota-associated genes with defined functions, to better understand the roles such genes play in the complicated ecosystem of the host intestine.

## Methods

### Bacterial strains

The indigenous *E. coli* strains termed typical *E. coli* (t*Ec*) and atypical *E. coli* (at*Ec*) were isolated from CD-1 mouse intestines. *V. cholerae* (*Vc*) O1 serotype N16961 (ref. 44) was used as a model pathogen in all experiments. All strains were routinely grown aerobically in Luria-Bertani (LB) broth (10 g tryptone, 5 g yeast extract, and 10 g NaCl per l) or on LB agar plates (15 g l^−1^ agar) at 37 °C. Streptomycin (200 μg ml^−1^) was added to selectively cultivate N16961.

### Construction of mutant and reporter strains

The at*Ec* Δ*ekatE* knockout mutant was created by allelic replacement as described previously^45^. The 500-base pair flanking sequences located at both ends were amplified by PCR with the primer sets listed in Supplementary Table 1 and used to introduce the mutation by homologous recombination. The primers used to construct the deletion mutant were designed according to the at*Ec* genomic sequence. A transcriptional luxCDABE reporter construct was constructed by PCR amplification of a 500-bp EcoRI–XbaI *ctxAB* promoter fragment from N16961 genomic DNA. This fragment was subsequently cloned into the EcoRI–XbaI sites of pVIK112, thereby generating pVIK112::P_*ctxAB*_. The transcriptional fusion reporter was then constructed by cloning the SalI–SacI promoterless *luxCDABE* fragment from pBBR-*lux* into pVIK112::P_*ctxAB*_ digested with the same enzymes, thus generating pVIK112::P_*ctxAB*_::*luxCDABE*. Chromosomal integration of the resultant fusion construct was performed as previously described^20^.

### Mouse models and housing conditions

Experiments were performed with CD-1 and Balb/c mice purchased from Orient Bio (Seoungnam, Korea). Pregnant Balb/c mice were purchased from Central Lab. Animal Inc. (Seoul, Korea) and raised for neonatal delivery. Mice were provided with irradiated food and sterile water. All animal studies were performed in compliance with the guidelines provided by the Department of Animal Resources of Yonsei Biomedical Research Institute. The Committee on the Ethics of Animal Experiments at the Yonsei University College of Medicine approved this study (permit number 2011-0166). For antibiotic treatment, 5- to 6-week-old CD-1 female mice were orally treated with streptomycin (1 mg), vancomycin (250 μg), or PBS as a control once a day for 7 days. Antibiotic doses were chosen to induce alteration of the gut microbiota composition without eliminating commensal microbes^11^. Bacterial counts were determined by plating serial dilutions of small intestine homogenates and faecal suspensions on plate count agar (Difco), LB agar, and Eosin Methylene Blue (EMB) agar plates. Fluorescence images of faecal suspensions were acquired with a LIVE/DEAD BacLight staining kit (Invitrogen)^46^. For *Vc* infection, antibiotic-treated CD-1 mice were orally inoculated with 10^7^ CFU of LB-grown N16961 cells after 18 h of food and water starvation. At 48 h postinfection, bacterial cells were recovered by homogenizing the mouse intestines in 2 ml of PBS containing 10% glycerol. The homogenates were diluted and plated on solid medium containing 200 μg ml^−1^ streptomycin. Luminescence was measured using a Victor X4 plate reader (Perkin Elmer). For experiments involving antibiotic cocktail treatment, mice were treated for 4 weeks with drinking water supplemented with four different antibiotics (ampicillin, 100 μg ml^−1^; vancomycin, 10 μg ml^−1^; metronidazole, 50 μg ml^−1^; and neomycin, 30 μg ml^−1^). For mouse intestinal transplantation, neonatal mice (4–5 days old) were treated orally once daily with 10^7^ CFU of t*Ec* or at*Ec* cells for 3 days. During the transplantation period, neonates were housed with their mothers.

### DNA purification from mouse faeces, small intestine and colon

Fresh stool pellets were collected before mice were killed. Faecal samples were stored at −80 °C before DNA purification. Immediately after the mice were euthanized, their small intestines and colons were recovered manually. Total genomic DNA was extracted from the faecal samples using a QIAamp DNA Stool Mini Kit (Qiagen) according to the manufacturer’s protocol. DNA was precipitated with ethanol and resuspended in 50 μl of TE buffer with 100 μg ml^−1^ RNase. Total genomic DNA was extracted from intestinal samples using the QIAamp DNA Stool Mini Kit (Qiagen) with a minor modification. Briefly, the recovered mouse organs were homogenized in 2 ml of PBS and centrifuged at low speed to remove tissue debris. The resultant supernatants were subjected to the purification procedures outlined in the QIAamp DNA Stool Mini Kit.

### 16S rRNA gene quantification by qRT–PCR

The relative abundance of each specific bacterial group was analysed by quantitative real-time PCR. Two microlitres of 10-fold diluted genomic DNA samples obtained from mouse faeces, small intestines, or colons served as the templates for SYBR Green-based quantitative PCR with reverse transcription (qRT–PCR) reactions. qRT–PCR was performed using a SYBR Premix Ex Taq kit (Takara Bio Inc., Japan) and gene-specific primers. All primers used in this study are listed in Supplementary Table 1. For each sample, at least three qRT–PCR replicates were performed. The total volume of each reaction was 25 μl. Each reaction contained DNA template (diluted 10- or 100-fold), 0.2 mM dNTPs, 1.5 mM MgCl_2_, 1.25 U SYBR premix Ex Taq DNA polymerase, 2.5 μl of 10 × PCR buffer, and 0.2 μM of each species-specific primer. Primers were designed to amplify the 16 S region as previously described^8^,^47^,^48^,^49^,^50^,^51^. Thermocycling conditions were as follows: 94 °C for 3 min, followed by 35 cycles (faecal samples) or 40 cycles (small intestine or colon samples) of 94 °C for 30 s, 60 or 62 °C for 45 s, and 72 °C for 1 min. The level of each gene was normalized to that of the host housekeeping gene *gapdh*. Results are expressed relative to the 16S rRNA level obtained with species-specific primers from an intestinal sample isolated from control mice.

### RAPD assay

*E. coli* strains isolated from antibiotic-treated mice were genotyped by random amplified polymorphic DNA (RAPD) fingerprinting as described elsewhere^52^. Briefly, genomic DNA was extracted using a G-Spin Genomic DNA Extraction Kit (iNtRON Biotechnology Inc.) following procedures provided by the manufacturer. A dendrogram was generated with XLSTAT software (Addinsoft USA, USA) based on the unweighted pair group method using an arithmetic algorithm (UPGMA).

### Whole-genome sequence and annotation

The t*Ec* and at*Ec* strains were grown in LB medium at 37 °C for 15–16 h, with shaking under aerobic conditions. Genomic DNA was extracted from bacteria using the G-Spin Genomic DNA Extraction Kit (iNtRON Biotechnology Inc.). Bacterial genomes were sequenced on an Illumina MiSeq system (Illumina, Inc., USA). Sequence reads (t*Ec*: 7,156,532 reads with a total read length of 2,281,359,270 bp; at*Ec*: 6,496,870 reads with a total read length of 1,721,860,260 bp) were assembled *de novo* using GS Assembler v. 2.6 (Roche Diagnostics) and CLC genomics workbench 6.0 (CLC bio, Denmark). This assembly resulted in 62 and 152 contigs for t*Ec* and at*Ec*, respectively. The contigs and PCR-based long reads were combined through manual curation using CodonCode Aligner 3.7.1 (CodonCode Corp., Dedham, MA, USA). The gaps within and between contigs were filled by custom primer walking and long-distance PCR amplification, followed by DNA sequencing with an ABI 3730XL sequencer. Coding sequences (CDSs) were predicted by Prodigal^53^ and tRNAs were searched using tRNAscan-SE^54^. The rRNAs and other noncoding RNAs were searched by a covariance model search with Rfam 12.0 database^55^. Basic genetic information such as GC content, number of ORFs, and prediction of ORF function was analysed by CLgenomics software (http://www.chunlab.com). The CDSs were classified into different groups based on their roles, with reference to orthologous groups (EggNOG 4.1; http://eggnogdb.embl.de)^56^. For more functional annotation, the predicted CDSs were compared with KEGG^57^ and SEED^58^ databases (BLASTP). The complete genome sequence of t*Ec* was 4,726,216 bp and its G+C content was 50.65%. Gene prediction identified 4,403 putative CDSs. The total genome length and G+C content of at*Ec* were 5,243,781 bp and 50.56%, respectively; 5,019 putative CDSs were identified in the at*Ec* genome.

### Bioinformatic analyses

Tetranucleotide frequency and CAI were calculated following procedures described elsewhere^59^,^60^. The pairwise overall similarity between genome sequences was calculated using the OrthoANI method^61^ and the dendrogram (shown in Supplementary Fig. 5) was generated using the UPGMA method. Sequences for phylogenetic analysis of KatG and KatE were retrieved from the GenBank database using the BLAST algorithm. Sequences were aligned with MUSCLE v3.8.31 (ref. 62) and aligned positions with >50% gaps were removed using GBLOCKS v0.91 (ref. 63). The phylogenetic relationships were inferred with RAxML v8.2.4 (ref. 64) and the trees (shown in Supplementary Fig. 7) were visualized using Dendroscope v3.2.2 (ref. 65). The trees were rooted by proteins that belong to the basal clade of each catalase group^66^.

### Catalase activity assays

A native gel-based catalase activity assay was performed as previously reported^67^,^68^. Briefly, proteins in bacterial lysates were resolved on a 7.5% nondenaturing acrylamide gel and stained with 3,3′-diaminobenzidine (Sigma). Lysates were prepared by sonicating bacterial cell suspensions in 50 mM Tris-HCl, pH 7.4.

### Construction of recombinant t*Ec* and *Vc* strains

A DNA element encompassing the *eKatE* gene and its own promoter was PCR amplified and cloned into the multicloning site of pBAD24 plasmid. The resultant plasmid, named pBAD24::*eKatE*, was transformed into the t*Ec* strain. PCR primers used for cloning are listed in Supplementary Table 1. A 600 bp non-functional region between the *VC0512* and *VC0513* genes in the genome of *Vc* N16961 strain was PCR amplified and ligated into pVIK112 plasmid. The resultant plasmid, pVIK112-600 bp, was digested with SacI and ligated with the SacI-digested PCR product that amplified the *eKatE* gene locus in the pBAD24::*eKatE* plasmid. The final plasmid, named pVIK112+*eKatE*, was conjugated into N16961. Transconjugants, in which pVIK112+*eKatE* was integrated in the 600 bp noncoding region via homologous recombination, were selected and verified by sequencing. pVIK112 with only the 600 bp non-functional sequence was used as a control. PCR primers used are listed in Supplementary Table 1.

### Measurement of ROS levels in intestinal extracts

Five to six neonatal mice were orally inoculated with 1 × 10^9^ CFU of t*Ec* and at*Ec* cells once a day for 3 days. The mice were killed and their intestinal contents were homogenized and resuspended in 2 ml of 0.1 M Tris/HCl, pH 7.5. Large particulates were removed by centrifugation at 1,000 r.p.m. for 5 min at 4 °C, after which the supernatants were harvested and incubated with 10 μM of the hydrogen peroxide-specific dye Peroxy Orange 1 (PO1, Tocris Bioscience) for 30 min in the dark. The level of orange fluorescence, which is indicative of the level of H_2_O_2_, was determined with a Victor X4 plate reader.

### BLASTp analyses of catalase proteins

To provide further information about the distribution of catalase proteins among commensal microbes in the human gut, we BLAST searched amino-acid sequences obtained from an integrated catalogue of reference genes in the human gut microbiome^69^. This comprehensive catalogue is composed of 9,879,896 non-redundant genes, which is a near complete set of genes found in almost all human gut bacteria. We also searched against an integrated gene catalogue of the mouse gut metagenome^70^ to outline the relative abundance of catalase proteins in human versus mouse intestine. The mouse metagenome catalogue comprises 2,572,074 non-redundant bacterial genes present in faecal samples collected from 184 mice of diverse backgrounds. We downloaded the amino-acid sequences listed in the companion web-servers and used those for pairwise sequence alignments using the BLASTp algorithm.

### Statistical analysis

Data are expressed as mean±standard error of the mean (SEM). Unpaired Student’s *t*-test and ANOVA (for Fig. 4d and Supplementary Fig. 1d-f) were used to determine whether differences between groups were significant. A *P* value<0.05 was considered to indicate statistical significance. All experiments were repeated for reproducibility.

### Data availability

The genomic sequences of the t*Ec* and at*Ec* strains have been deposited in the NCBI genome database with accession codes LRAB00000000 and LRBX00000000, respectively. The authors declare that all other data supporting the findings of this study are available within the article and its Supplementary Information files.

## Additional information

**How to cite this article:** Yoon, M. Y. *et al*. A single gene of a commensal microbe affects host susceptibility to enteric infection. *Nat. Commun.* 7:11606 doi: 10.1038/ncomms11606 (2016).

## Supplementary Material

Supplementary Information Supplementary Figures 1-12 and Supplementary Tables 1-4

Supplementary Data 1 Metagenome catalog search results
