# Supplementary material for: A single gene of a commensal microbe affects host susceptibility to enteric infection
Source: Nat Commun. 2016 May 13;7:11606. doi: 10.1038/ncomms11606 (PMC5482719; doi:10.1038/ncomms11606)
Supplement: Supplementary Information — Supplementary Figures 1-12 and Supplementary Tables 1-4 [file ncomms11606-s1.pdf]

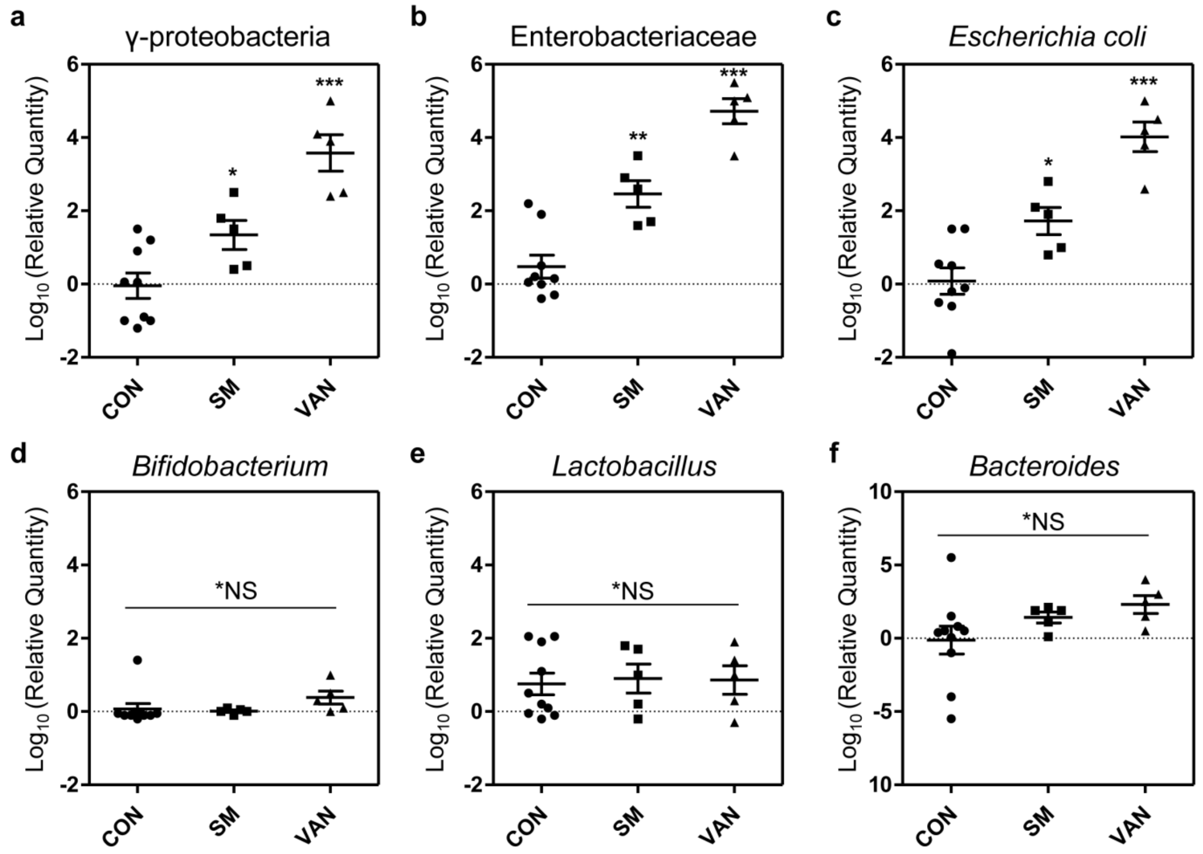

Supplementary Figure 1. Relative quantities of the indicated groups of bacteria in SI tissue homogenates. Adult mice (n=5) were treated with SM or VAN as described in Fig. 1A. Untreated mice were used as controls. The copy numbers of the 16S rRNA gene of γ-proteobacteria (**a**), Enterobacteriaceae (**b**), *E. coli* (**c**), *Bifidobacterium* (**d**), *Lactobacillus* (**e**) and *Bacteroides* (**f**) were determined by real-time PCR and normalized to that of host *gapdh*. Values are expressed as means ± SEMs and are displayed on a log scale. *$P < 0.05$, **$P < 0.005$, ***$P < 0.001$ vs. the relative quantities of the control group for each panel. *NS, not significantly different between the groups as determined by ANOVA.

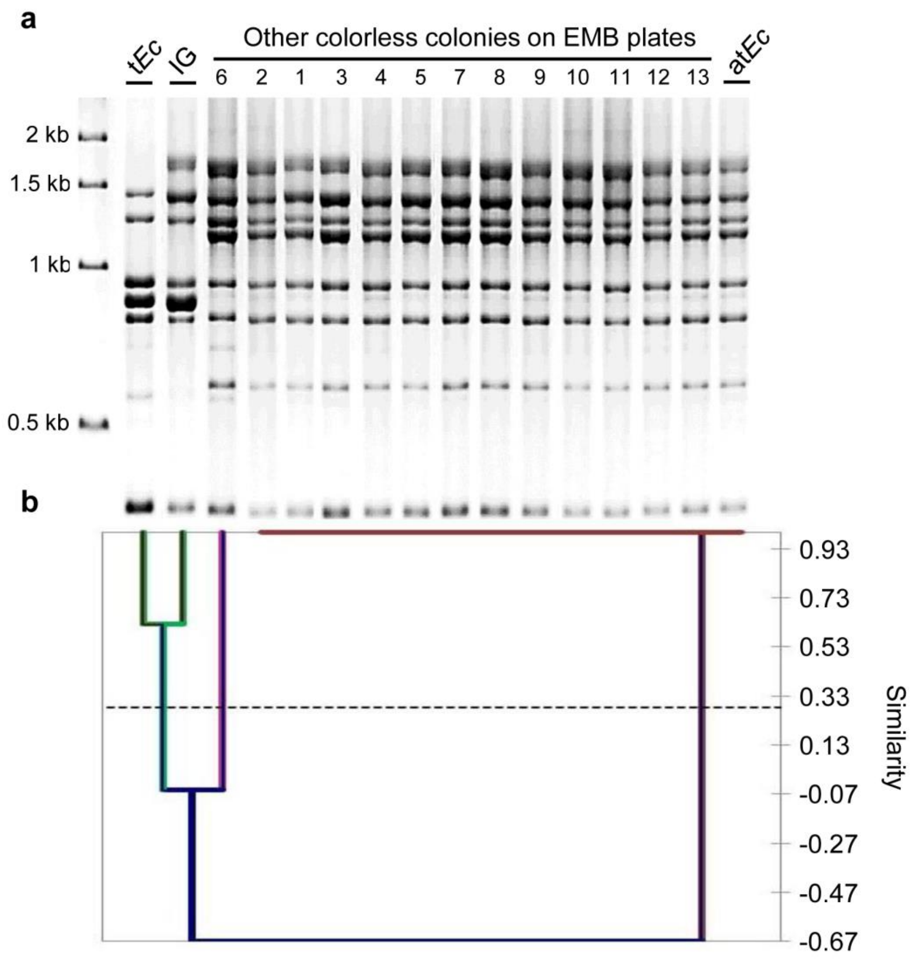

Supplementary Figure 2. Genome typing of *E. coli* cells growing as colorless colonies. (**a**) RAPD amplification products. The RAPD reaction was performed as described in Methods with the primers listed in Supplementary Table 1. Reaction products were analyzed on agarose gels. A total of 14 colorless colonies (including "at*Ec*" in the far right lane; the genome of this strain was fully sequenced) were included in the assay. In addition, one colony exhibiting the typical *E. coli* growth phenotype on EMB plates ("t*Ec*") and one intermediate green colony isolated from the feces of VAN-treated mice ("IG") were analyzed. (**b**) Dendrogram based on RAPD analysis. Thirteen of the colorless strains, including at*Ec*, shared 100% genomic similarity. However, strain 6 was an exception.

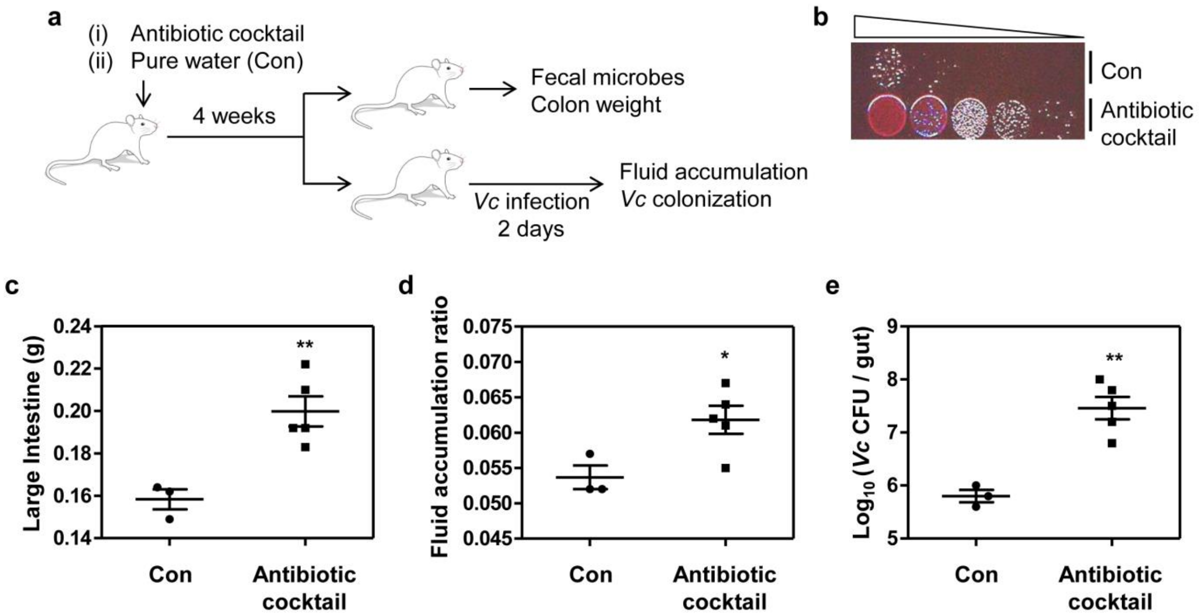

Supplementary Figure 3. Atypical *E. coli* cells proliferate in mice treated with a mixture of 4 antibiotics. (**a**) Schematic diagram of the experimental procedure. Four-week-old female Balb/c mice were treated with an antibiotic cocktail (ampicillin, 100 µg/mL; vancomycin, 10 µg/mL; metronidazole, 50 µg/mL; and neomycin, 30 µg/mL) for 4 weeks. Antibiotics were administered in the drinking water. (**b**) Representative EMB plate showing substantial proliferation of colorless atypical *E. coli* strains after antibiotic treatment. Aliquots of mouse fecal suspensions were serially diluted and spotted onto EMB plates. (**c**) The large intestine was removed from each control (n=3) or antibiotic-treated mouse (n=5) and weighed. Values are expressed as means ± SEMs and are displayed on a linear scale. $^{**}P < 0.01$ vs. control mice. (**d**) Antibiotic-treated (n=5) and control (n=3) mice were challenged with *Vc*. On day 2 post-infection, the fluid accumulation (FA) ratio of each group was measured and is plotted on a linear scale. $^{*}P < 0.05$ vs. the FA ratio of the *Vc*-infected control group. (**e**) *Vc* colonization in each group. The number of *Vc* CFUs was determined by plating serial dilutions of intestinal homogenates onto LB plates supplemented with 200 µg/mL SM. Values are expressed as means ± SEMs and are displayed on a log scale. $^{*}P < 0.005$ vs. *Vc* CFUs from the control group.

a. Genome information

	size	GC contents	CDS
**t*Ec***	**4.72 Mb**	**50.65 %**	**4,403**
**at*Ec***	**5.24 Mb**	**50.56 %**	**5,019**
E. coli K12	4.64 Mb	50.79 %	4,299

b. MUMmer dot plot

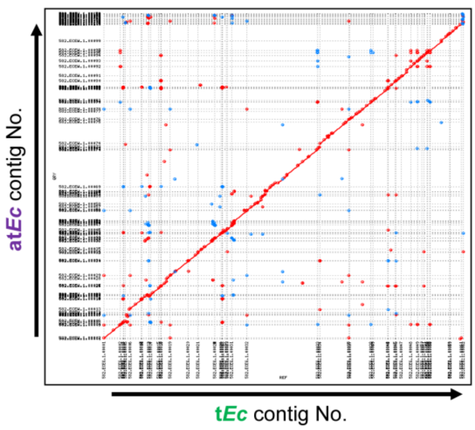

c. Genetic structure of *lac* operon

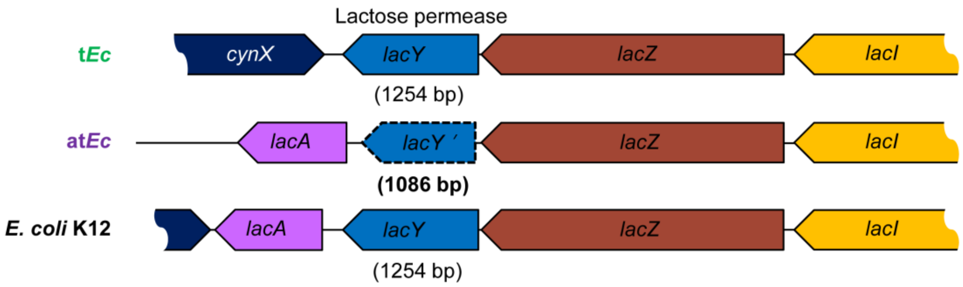

Supplementary Figure 4. Whole genome sequencing provides an explanation for why the at*Ec* strain forms colorless colonies on EMB plates. (**a**) Comparison of the genome sequences of the at*Ec*, t*Ec*, and K12 strains. (**b**) Alignment of the at*Ec* and t*Ec* genomes using the Maximal Unique Match (MUM) system, which extracts the longest possible nucleotide matches of both genomes. Each dot represents a match between the two genomes. Red and blue dots indicate matches in the forward and reverse direction, respectively. (**c**) Comparison of the genetic structures of the *lac* operons of the different strains. The *lacY* gene of the at*Ec* strain was truncated (dotted line), while the t*Ec* strain harbored an intact *lacY* gene. The length and direction of each arrow indicate the relative size and transcriptional direction of each gene.

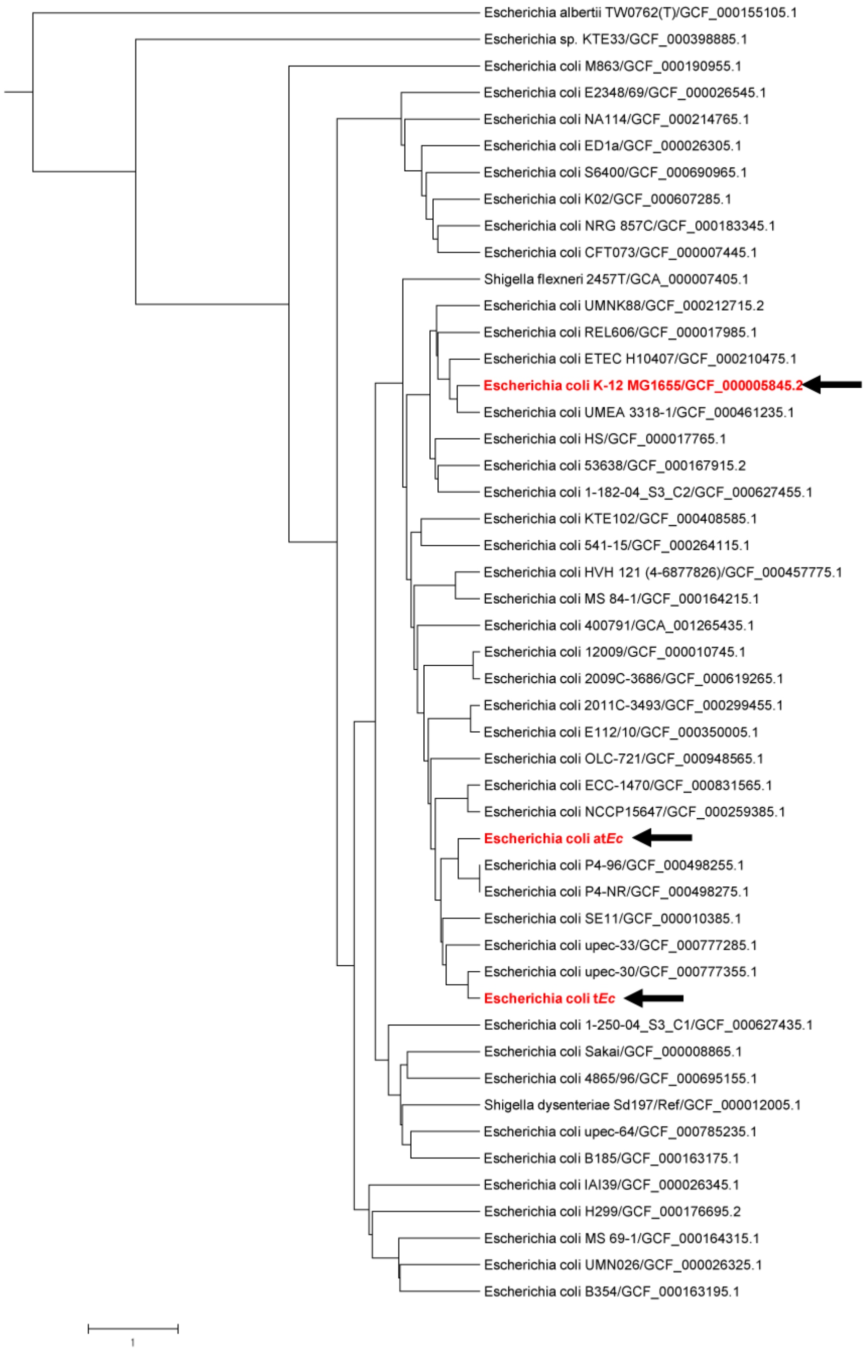

Supplementary Figure 5. Genome-based phylogenetic relationship of at*Ec*, t*Ec* strains with other representative *E. coli* and *Shigella* strains. Three *E. coli* strains, whose genomes were compared in Fig. S4A, were indicated with black arrows. The dendrogram was constructed based on average nucleotide identity (ANI) values.

a. Catalase genes

	KatE	KatG	extra KatE (eKatE)
t*Ec*	753 aa (750/753)	726 aa (725/726)	
at*Ec*	753 aa (749/753)	726 aa (725/726)	**750 aa**
E. coli K12	753 aa	726 aa	

b

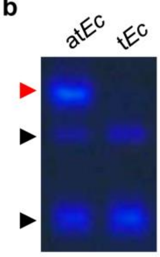

c. Amino acid sequence alignment of eKatE and KatE

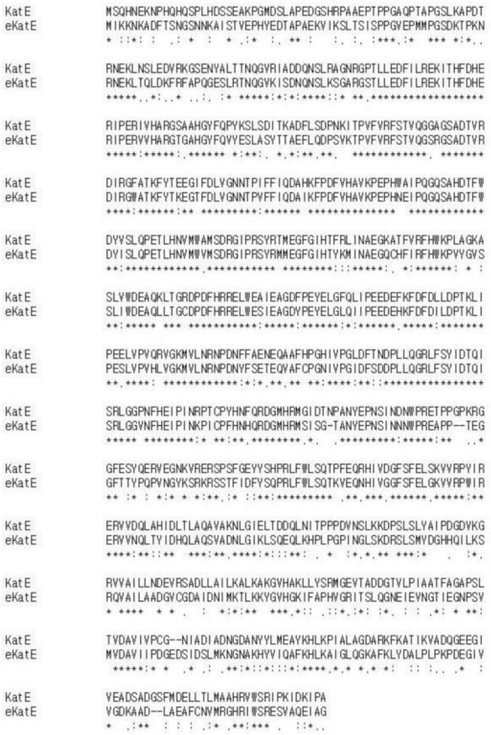

d. Nucleotide sequence identity of at*Ec* *eKatE* gene

	***E. coli* atEc**	***E. coli* K02**	**Serratia sp. SCBI**	**Frankia sp. BMG5.23**
E. coli at*Ec*		0/2253*	160/2250	160/2250
E. coli K02	100%		160/2250	160/2250
Serratia sp. SCBI	92.89%	92.89%		0/2250
Frankia sp. BMG5.23	92.89%	92.89%	100%	

* No. of different nucleotides/No. of nucleotides compared

Supplementary Figure 6. The at*Ec* strain harbors an extra catalase-encoding gene. (**a**) The at*Ec* strain possesses three catalase genes, while the t*Ec* and K12 strains have two. The amino acid (aa) length of each catalase is indicated and the number of identical residues compared to the corresponding K12 protein is shown in parentheses. (**b**) In-gel catalase activity assay. Proteins in bacterial extracts were separated by native gel electrophoresis and catalase-specific bands were stained as described in the Methods section. Three distinct bands are shown in the left lane of the gel (at*Ec* cell lysate). (**c**) Amino acid sequence alignment of KatE and eKatE. (**d**) The top 3 genes exhibiting the highest sequence identities to the *eKatE* gene*.* To identify similar genes, the NCBI database was searched using the BLASTn algorithm.

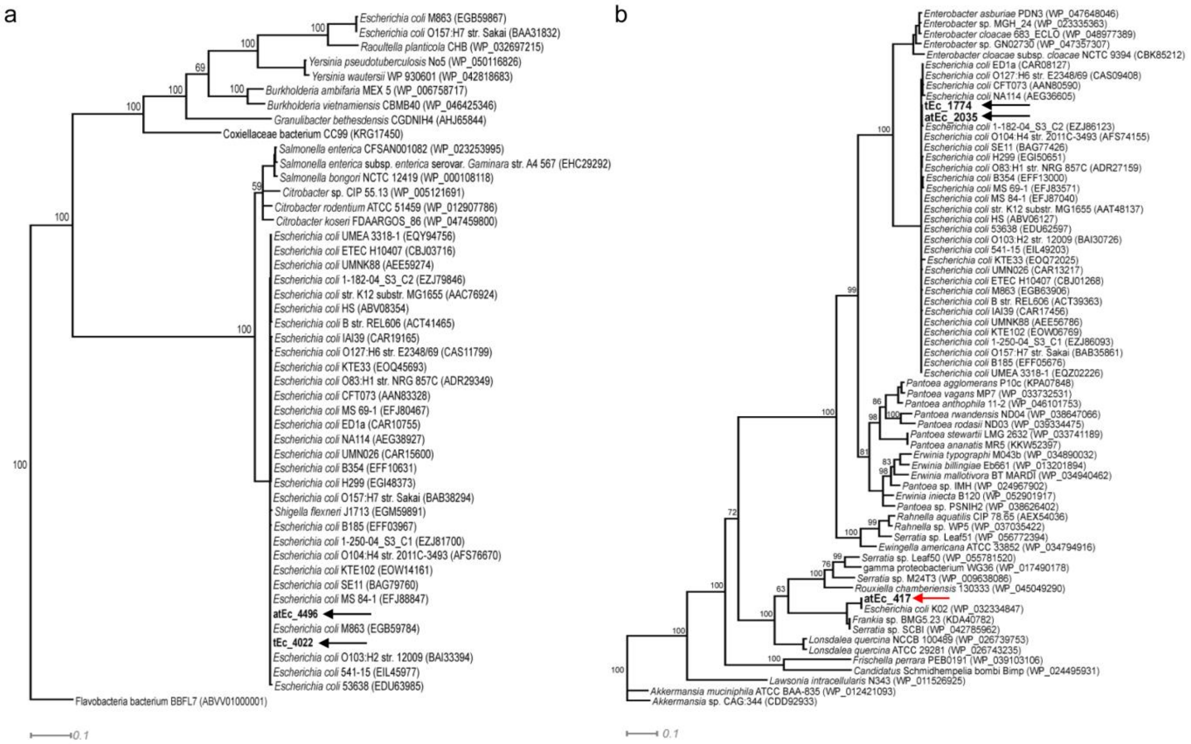

Supplementary Figure 7. Maximum likelihood trees of *E. coli* and related taxa constructed using the amino acid alignments of KatG (a) and KatE (b) proteins. Numbers above branches show maximum-likelihood bootstrap supports from 1,000 non-parametric replicates (shown only if they were > 50). The trees were rooted by outgroups *Flavobacteria bacterium* BBFL7 (**a**) and *Akkermansia* sp. CAG:344 (**b**). Accession numbers of protein sequences are indicated between parentheses. The scale represents the number of substitutions per site. KatG and KatE proteins from at*Ec* and t*Ec* strains are shown with black arrows. The eKatE protein from at*Ec* strain is shown with red arrow.

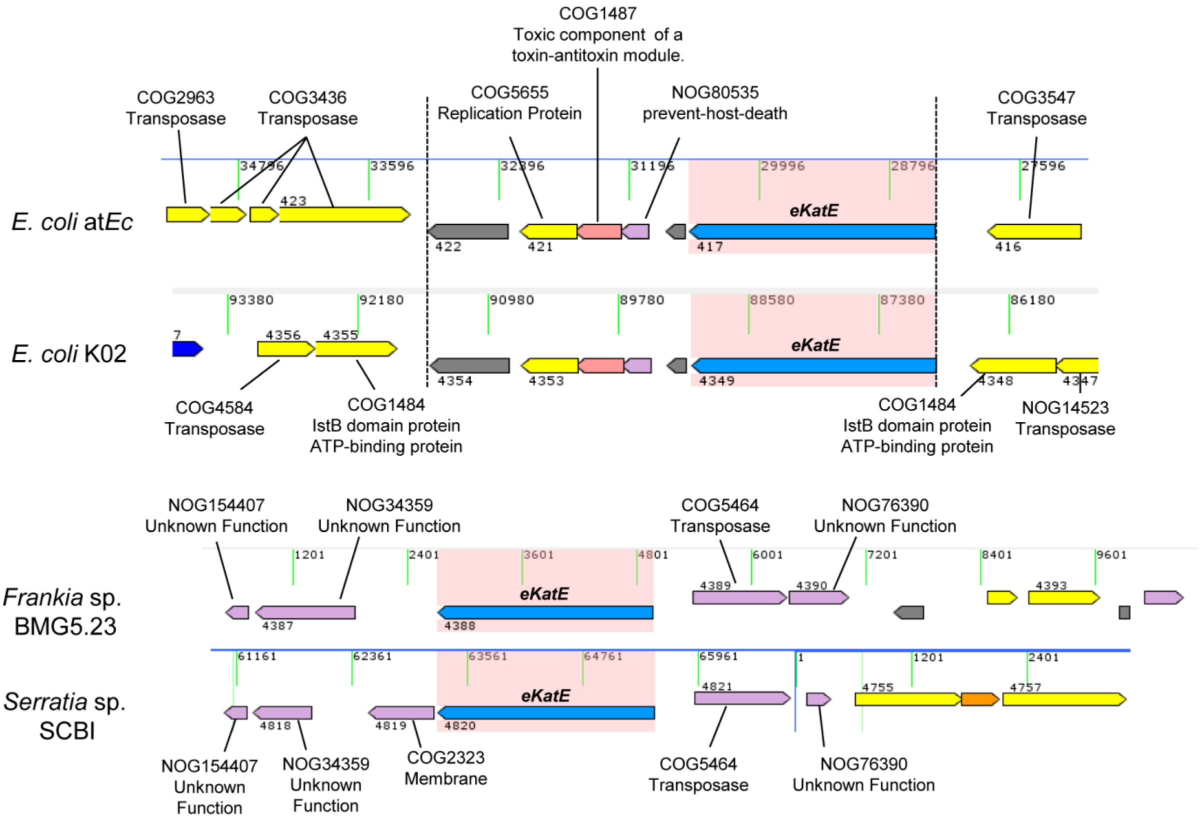

Supplementary Figure 8. Synteny comparison of *eKatE* genetic loci among at*Ec*, K02, *Frankia* sp. BMG5.23 and *Serratia* sp. SCBI. The *eKatE* genes (marked in blue arrows) and neighboring genes with annotation are displayed in each genome. Regions shown between two vertical dotted lines are identical in at*Ec* and K02 strains.

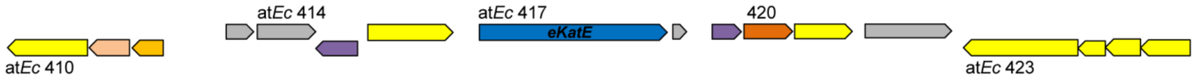

Gene	Size (bp)	Function
at*Ec* 410	807	resolvase, site-specific recombinase XerD
at*Ec* 411	306	CcdB
at*Ec* 412	219	plasmid maintenance protein CcdA
at*Ec* 413	258	hypothetical protein
at*Ec* 414	591	hypothetical protein
at*Ec* 415	348	hypothetical protein
at*Ec* 416	855	transposase
**at*Ec* 417**	**2,253**	**eKatE**
at*Ec* 418	162	hypothetical protein
at*Ec* 419	255	prevent-host-death
at*Ec* 420	423	toxic component of a toxin-antitoxin (TA) module
at*Ec* 421	513	replication Protein
at*Ec* 422	729	hypothetical protein
at*Ec* 423	1,260	transposase
at*Ec* 424	249	transposase
at*Ec* 425	348	transposase
at*Ec* 426	381	transposase

Supplementary Figure 9. A high-resolution physical map of the *eKatE* gene locus in the at*Ec* chromosome. In the table, gene numbers, gene sizes (in bp) and putative functions are presented. Genes involved in transposition are shown with gray highlights, while those involved in plasmid maintenance are shown with orange highlights.

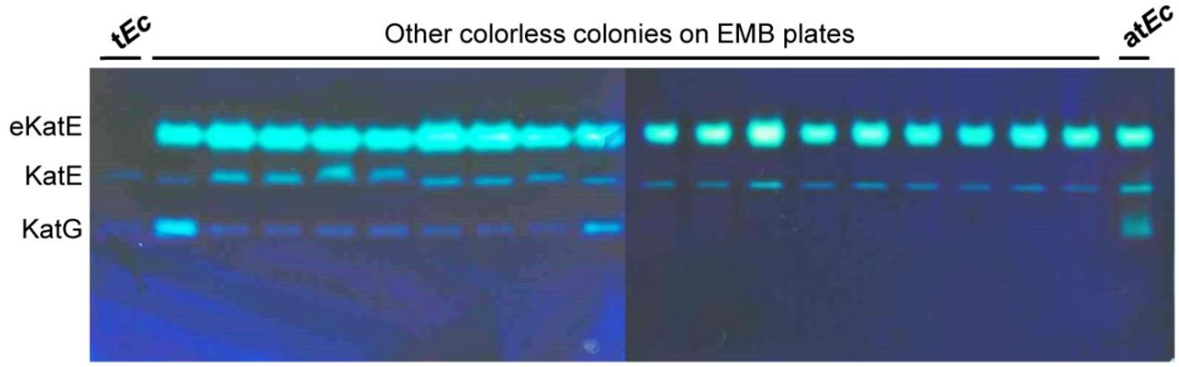

Supplementary Figure 10. Native gel-based catalase assay. All other *E. coli* strains (n=18) that formed colorless colonies on EMB plates produced eKatE. The native gel-based catalase activity assay was performed as described in Fig. 5A.

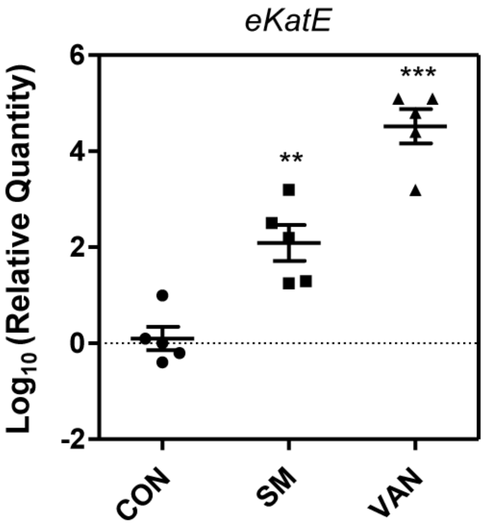

Supplementary Figure 11. Relative quantities of the *eKatE* gene present in SI tissue homogenates (n=5) as determined by real-time PCR. The level of mouse *gapdh* in each sample was used for normalization. The ratios of *eKatE* to host *gapdh* are displayed on a log scale (means ± SEMs). **P < 0.005, ***P < 0.001 vs. the control group.

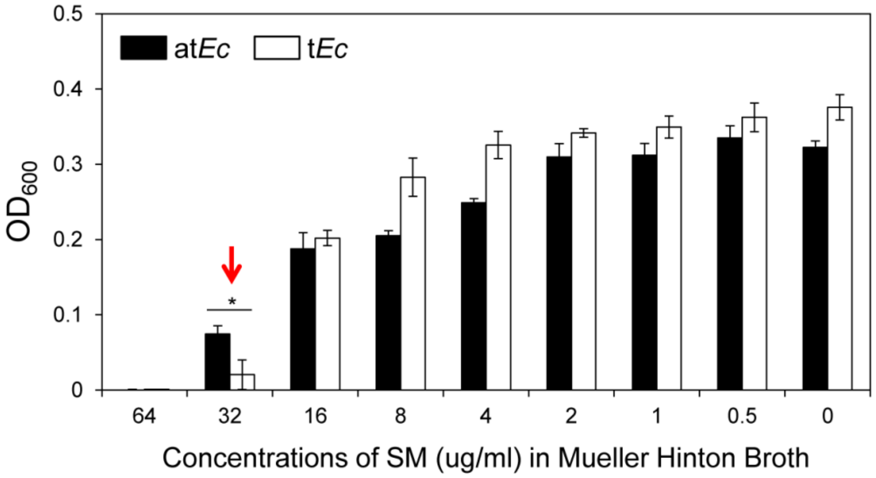

Supplementary Figure 12. Bacterial growth of at*Ec* and t*Ec* strains in the presence of increasing amounts of SM. Strains were grown in Mueller Hinton Broth, which has been commonly used for antibiotic sensitivity assay. *$P < 0.05$ between two growths.

Supplementary Table 1. Primers used in this study

1. Cloning		
Deletion of *eKatE* gene in at*Ec* (5' to 3')*		
left-flanking region		F: ATATAGAGCTCGATGATATGAACGGCTCACCGG
		R: TAAATGGATCCCCTTTTCGGCAGGTGCAGTATC
right-flanking region		F: CCCTTGGATCCATGATGCGTTGCCGTTGCCCAA
		R: ACACGAGCTCGACTTGTGTAGTAACGTTACTACG
Construction of P_{ctxAB}::*luxCDABE* transcriptional fusion (5' to 3')		
P_{ctxAB}		F: CACATGAATTCACTATCGAGTCAGAGCAATCCG
		R: ATTGGTCTAGATTGTTTAACAGAAAAATAATTGATCAAAAC
luxCDABE		F: ATTCGTCGACACTAAAAAAATTTCATTCATTATT
		R: GATATGAGCTCTACTCAGGAGAGCGTTCAC
Construction of *Vc* N16961 strain containing chromosomally encoded *ekatE* gene		
at*Ec* *ekatE*		F:CATATGAATTCGGCAAGCCCACTTATCGTCAA
		R: CAATTAAGCTTTTAGCCAGCAATTTCCTGTGCT
VC0512 600bp		F: AATTCGAATTCTGAGGGAGCACTCCTGATAAG
		R: GATATGAGCTCCTGAGGCATCCATATCGGAAC
Construction of t*Ec* strain that express the *ekatE* gene		
pBAD24::*ekatE*		F: AATTCGAGCTCGGCAAGCCCACTTATCGTCAA
		R: ATATCGAGCTCTGTCTTACAGGTGGATGGCCT
2. Quantitative real-time PCR		
Organisms	Target Genes	Sequences (5' to 3')
Mus musculus	*gapdh*	F: GTGTTCCTACCCCCAATGTGT
		R: ATTGTCATACCAGGAAATGAGCTT
γ-Proteobacteria	*16S rRNA*	F: C**M**ATGCCGCGTGTGTGAA**
		R: ACTCCCCAGGCGGTC**D**ACTTA***
Enterobacteriaceae	*16S rRNA*	F: GTTAATACCTTTGCTCATTGA
		R: ACCAGGGTATCTAATCCTGTT
E. coli	*16S rRNA*	F: CAGCCACACTGGAACTGAGA
		R: GTTAGCCGGTGCTTCTTCTG
Bacteroides	*16S rRNA*	F: AACGCTAGCTACAGGCTT
		R: CAATCGGAGTTCTTCGTG
Lactobacillus	*16S rRNA*	F: AGCAGTAGGGAATCTTCCA
		R: CACCGCTACACATGGAG
Bifidobacterium	*16S rRNA*	F: TCGCGTCYGGTGTGAAAG
		R: CCACATCCAGCRTCCAC
Vibrio cholerae	*16S rRNA*	F: CTGGAACTGAGACACGGTCC
		R: CATGCGCTTTACGCCCAGTA
at*Ec*	*eKatE*	F: GAGTCTTTGGTTCCCGTTCA
		R: ACTCCTCCCAAACGGCTAAT
3. RAPD		
M13 primer		5'- GAGGGTGGCGGTTCT

F, forward; R, reverse.
* Restriction enzyme recognition sequences are underlined.
**M is either A or C.
***D is either G, A or T.

Supplementary Table 2. Tetranucleotide frequency correlation coefficients (TETRA) values calculated between *katE* genes of at*Ec*, t*Ec* and representative *E. coli* strains.

	Strains	1	2	3	4	5	6	7	8	9	10	11	12	13	14	15	16	17	18	19	20	21	22	23	24	25	26	27	28	29	30	31	32	33	34	35	36
1	E. coli O157:H7 str. Sakai	1	0.95	0.907	0.928	0.913	0.956	0.878	0.956	0.952	0.933	0.929	0.887	0.931	0.928	0.88	0.887	0.968	0.905	0.946	0.901	0.907	0.773	0.23	0.948	0.934	0.952	0.933	0.973	0.246	0.967	0.932	0.161	0.253	0.949	0.105	0.95
2	E. coli UMN026	0.95	1	0.916	0.901	0.899	0.94	0.885	0.94	0.936	0.927	0.925	0.898	0.904	0.935	0.88	0.898	0.93	0.929	0.935	0.915	0.896	0.782	0.231	0.929	0.905	0.931	0.927	0.933	0.278	0.925	0.922	0.192	0.282	0.929	0.096	0.925
3	E. coli IAI39	0.907	0.916	1	0.896	0.95	0.905	0.865	0.905	0.9	0.904	0.915	0.877	0.899	0.907	0.873	0.877	0.897	0.909	0.898	0.915	0.941	0.802	0.193	0.898	0.901	0.909	0.904	0.897	0.258	0.886	0.902	0.149	0.268	0.896	0.056	0.895
4	E. coli str. K-12 substr. MG1655	0.928	0.901	0.896	1	0.91	0.917	0.822	0.917	0.914	0.942	0.943	0.831	0.997	0.941	0.831	0.831	0.933	0.882	0.939	0.897	0.912	0.785	0.191	0.929	0.998	0.937	0.942	0.928	0.255	0.929	0.942	0.153	0.257	0.927	0.115	0.923
5	E. coli O83:H1 str. NRG 857C	0.913	0.899	0.95	0.91	1	0.896	0.893	0.896	0.892	0.896	0.902	0.906	0.91	0.902	0.901	0.906	0.9	0.909	0.913	0.927	0.993	0.813	0.206	0.891	0.912	0.911	0.896	0.904	0.245	0.894	0.894	0.172	0.258	0.888	0.025	0.891
6	E. coli O104:H4 str. 2011C-3493	0.956	0.94	0.905	0.917	0.896	1	0.834	1	0.996	0.955	0.954	0.849	0.923	0.944	0.839	0.849	0.943	0.879	0.968	0.874	0.892	0.778	0.231	0.989	0.922	0.925	0.955	0.948	0.288	0.934	0.955	0.214	0.295	0.991	0.039	0.986
7	E. coli CFT073	0.878	0.885	0.865	0.822	0.893	0.834	1	0.834	0.829	0.834	0.836	0.983	0.825	0.849	0.983	0.983	0.85	0.919	0.833	0.91	0.885	0.778	0.238	0.829	0.826	0.868	0.834	0.851	0.215	0.842	0.831	0.139	0.232	0.835	0.073	0.834
8	E. coli SE11	0.956	0.94	0.905	0.917	0.896	1	0.834	1	0.996	0.955	0.954	0.849	0.923	0.944	0.839	0.849	0.943	0.879	0.968	0.874	0.892	0.778	0.231	0.989	0.922	0.925	0.955	0.948	0.288	0.934	0.955	0.214	0.295	0.991	0.039	0.986
9	E. coli O103:H2 str. 12009	0.952	0.936	0.9	0.914	0.892	0.996	0.829	0.996	1	0.952	0.951	0.847	0.92	0.941	0.834	0.847	0.943	0.877	0.966	0.873	0.887	0.774	0.22	0.987	0.919	0.921	0.952	0.948	0.281	0.933	0.952	0.208	0.287	0.989	0.043	0.986
10	E. coli HS	0.933	0.927	0.904	0.942	0.896	0.955	0.834	0.955	0.952	1	0.991	0.849	0.946	0.987	0.852	0.849	0.916	0.893	0.937	0.889	0.896	0.764	0.224	0.965	0.947	0.922	1	0.917	0.278	0.908	0.998	0.183	0.284	0.963	0.069	0.956
11	E. coli B str. REL606	0.929	0.925	0.915	0.943	0.902	0.954	0.836	0.954	0.951	0.991	1	0.853	0.946	0.983	0.854	0.853	0.909	0.901	0.934	0.893	0.902	0.766	0.221	0.964	0.947	0.918	0.991	0.91	0.264	0.902	0.99	0.185	0.27	0.962	0.048	0.955
12	E. coli O127:H6 str. E2348/69	0.887	0.898	0.877	0.831	0.906	0.849	0.983	0.849	0.847	0.849	0.853	1	0.834	0.862	0.976	1	0.861	0.927	0.843	0.922	0.897	0.781	0.222	0.842	0.835	0.876	0.849	0.861	0.194	0.852	0.846	0.128	0.209	0.846	0.085	0.846
13	E. coli ETEC H10407	0.931	0.904	0.899	0.997	0.91	0.923	0.825	0.923	0.92	0.946	0.946	0.834	1	0.944	0.833	0.834	0.937	0.882	0.942	0.899	0.913	0.783	0.189	0.934	0.999	0.937	0.946	0.931	0.254	0.932	0.946	0.155	0.256	0.933	0.113	0.928
14	E. coli UMNK88	0.928	0.935	0.907	0.941	0.902	0.944	0.849	0.944	0.941	0.987	0.983	0.862	0.944	1	0.863	0.862	0.914	0.905	0.938	0.888	0.901	0.778	0.229	0.955	0.945	0.923	0.987	0.911	0.263	0.906	0.985	0.165	0.268	0.952	0.061	0.948
15	E. coli NA114	0.88	0.88	0.873	0.831	0.901	0.839	0.983	0.839	0.834	0.852	0.854	0.976	0.833	0.863	1	0.976	0.85	0.919	0.835	0.918	0.893	0.766	0.214	0.835	0.835	0.869	0.852	0.852	0.199	0.841	0.848	0.126	0.213	0.84	0.09	0.837
16	E. coli ED1a	0.887	0.898	0.877	0.831	0.906	0.849	0.983	0.849	0.847	0.849	0.853	1	0.834	0.862	0.976	1	0.861	0.927	0.843	0.922	0.897	0.781	0.222	0.842	0.835	0.876	0.849	0.861	0.194	0.852	0.846	0.128	0.209	0.846	0.085	0.846
17	E. coli B185	0.968	0.93	0.897	0.933	0.9	0.943	0.85	0.943	0.943	0.916	0.909	0.861	0.937	0.914	0.85	0.861	1	0.893	0.946	0.896	0.894	0.776	0.237	0.938	0.936	0.932	0.916	0.99	0.272	0.982	0.917	0.176	0.278	0.94	0.112	0.944
18	E. coli B354	0.905	0.929	0.909	0.882	0.909	0.879	0.919	0.879	0.877	0.893	0.901	0.927	0.882	0.905	0.919	0.927	0.893	1	0.876	0.967	0.903	0.797	0.22	0.878	0.884	0.89	0.893	0.888	0.231	0.885	0.889	0.128	0.246	0.88	0.082	0.881
19	E. coli MS 84-1	0.946	0.935	0.898	0.939	0.913	0.968	0.833	0.968	0.966	0.937	0.934	0.843	0.942	0.938	0.835	0.843	0.946	0.876	1	0.882	0.909	0.788	0.222	0.964	0.94	0.935	0.937	0.948	0.279	0.939	0.936	0.199	0.287	0.962	0.064	0.961
20	E. coli MS 69-1	0.901	0.915	0.915	0.897	0.927	0.874	0.91	0.874	0.873	0.889	0.893	0.922	0.899	0.888	0.918	0.922	0.896	0.967	0.882	1	0.926	0.797	0.213	0.878	0.899	0.898	0.889	0.888	0.213	0.877	0.883	0.117	0.225	0.878	0.109	0.881
21	E. coli H299	0.907	0.896	0.941	0.912	0.993	0.892	0.885	0.892	0.887	0.896	0.902	0.897	0.913	0.901	0.893	0.897	0.894	0.903	0.909	0.926	1	0.81	0.211	0.893	0.914	0.903	0.896	0.895	0.247	0.884	0.894	0.174	0.257	0.889	0.037	0.891
22	E. coli M863	0.773	0.782	0.802	0.785	0.813	0.778	0.778	0.778	0.774	0.764	0.766	0.781	0.783	0.778	0.766	0.781	0.776	0.797	0.788	0.797	0.81	1	0.148	0.774	0.787	0.8	0.764	0.779	0.245	0.761	0.76	0.164	0.259	0.776	0.07	0.781
23	E. coli KTE33	0.23	0.231	0.193	0.191	0.206	0.231	0.238	0.231	0.22	0.224	0.221	0.222	0.189	0.229	0.214	0.222	0.237	0.22	0.222	0.213	0.211	0.148	1	0.234	0.197	0.217	0.224	0.215	0.652	0.241	0.229	0.686	0.658	0.233	0.041	0.237
24	E. coli KTE102	0.948	0.929	0.898	0.929	0.891	0.989	0.829	0.989	0.987	0.965	0.964	0.842	0.934	0.955	0.835	0.842	0.938	0.878	0.964	0.878	0.893	0.774	0.234	1	0.934	0.919	0.965	0.939	0.281	0.927	0.964	0.204	0.286	0.996	0.059	0.991
25	E. coli UMEA 3318-1	0.934	0.905	0.901	0.998	0.912	0.922	0.826	0.922	0.919	0.947	0.947	0.835	0.999	0.945	0.835	0.835	0.936	0.884	0.94	0.899	0.914	0.787	0.197	0.934	1	0.94	0.947	0.931	0.257	0.933	0.946	0.157	0.26	0.932	0.113	0.927
26	E. coli UPCE 64/2083	0.952	0.931	0.909	0.937	0.911	0.925	0.868	0.925	0.921	0.922	0.918	0.876	0.937	0.923	0.869	0.876	0.932	0.89	0.935	0.898	0.903	0.8	0.217	0.919	0.94	1	0.922	0.938	0.246	0.928	0.919	0.153	0.251	0.919	0.117	0.919
27	E. coli 53638	0.933	0.927	0.904	0.942	0.896	0.955	0.834	0.955	0.952	1	0.991	0.849	0.946	0.987	0.852	0.849	0.916	0.893	0.937	0.889	0.896	0.764	0.224	0.965	0.947	0.922	1	0.917	0.278	0.908	0.998	0.183	0.284	0.963	0.069	0.956
28	E. coli O145:H28 str. 4865/96	0.973	0.933	0.897	0.928	0.904	0.948	0.851	0.948	0.948	0.917	0.91	0.861	0.931	0.911	0.852	0.861	0.99	0.888	0.948	0.888	0.895	0.779	0.215	0.939	0.931	0.938	0.917	1	0.256	0.985	0.919	0.17	0.264	0.942	0.095	0.945
29	E. coli 541-15	0.246	0.278	0.258	0.255	0.245	0.288	0.215	0.288	0.281	0.278	0.264	0.194	0.254	0.263	0.199	0.194	0.272	0.231	0.279	0.213	0.247	0.245	0.652	0.281	0.257	0.246	0.278	0.256	1	0.27	0.28	0.812	0.987	0.28	-0.02	0.269
30	E. coli 1-250-04_S3_C1	0.967	0.925	0.886	0.929	0.894	0.934	0.842	0.934	0.933	0.908	0.902	0.852	0.932	0.906	0.841	0.852	0.982	0.885	0.939	0.877	0.884	0.761	0.241	0.927	0.933	0.928	0.908	0.985	0.27	1	0.911	0.18	0.277	0.93	0.1	0.936
31	E. coli 1-182-04_S3_C2	0.932	0.922	0.902	0.942	0.894	0.955	0.831	0.955	0.952	0.998	0.99	0.846	0.946	0.985	0.848	0.846	0.917	0.889	0.936	0.883	0.894	0.76	0.229	0.964	0.946	0.919	0.998	0.919	0.28	0.911	1	0.183	0.286	0.964	0.069	0.957
32	E. coli S6400	0.161	0.192	0.149	0.153	0.172	0.214	0.139	0.214	0.208	0.183	0.185	0.128	0.155	0.165	0.126	0.128	0.176	0.128	0.199	0.117	0.174	0.164	0.686	0.204	0.157	0.153	0.183	0.17	0.812	0.18	0.183	1	0.823	0.201	-0.07	0.186
33	E. coli 400791	0.253	0.282	0.268	0.257	0.258	0.295	0.232	0.295	0.287	0.284	0.27	0.209	0.256	0.268	0.213	0.209	0.278	0.246	0.287	0.225	0.257	0.259	0.658	0.286	0.26	0.251	0.284	0.264	0.987	0.277	0.286	0.823	1	0.286	-0.02	0.276
34	E. coli tEc	0.949	0.929	0.896	0.927	0.888	0.991	0.835	0.991	0.989	0.963	0.962	0.846	0.933	0.952	0.84	0.846	0.94	0.88	0.962	0.878	0.889	0.776	0.233	0.996	0.932	0.919	0.963	0.942	0.28	0.93	0.964	0.201	0.286	1	0.059	0.995
35	E. coli atEc ekatE	0.105	0.096	0.056	0.115	0.025	0.039	0.073	0.039	0.043	0.069	0.048	0.085	0.113	0.061	0.09	0.085	0.112	0.082	0.064	0.109	0.037	0.07	0.041	0.059	0.113	0.117	0.069	0.095	-0.02	0.1	0.069	-0.07	-0.02	0.059	1	0.05
36	E. coli atEc	0.95	0.925	0.895	0.923	0.891	0.986	0.834	0.986	0.986	0.956	0.955	0.846	0.928	0.948	0.837	0.846	0.944	0.881	0.961	0.881	0.891	0.781	0.237	0.991	0.927	0.919	0.956	0.945	0.269	0.936	0.957	0.186	0.276	0.995	0.05	1

TETRA values of *eKatE* gene against each of other *E. coli katE* genes were shown in the row highlighted in yellow. Four *katE* genes that show relatively low TETRA values were also indicated with gray higlights.

Supplementary Table 3. Codon adaptation index (CAI) and GC contents of catalase genes in at*Ec* strain.

Genes	Size (bp)	CAI	%G+C
katE	2,262	0.751	51.5
katG	2,181	0.812	55.8
eKatE	2,253	**0.671**	**43.6**

Supplementary Table 4. Genes involved in oxidative stress responses

Genes in at*Ec*	Genes in t*Ec*	Function	eggNOG ID
at*Ec*_0008	t*Ec*_4463	Aerobic respiration control protein arcA	COG0745
at*Ec*_0025	t*Ec*_0012	Hydrogen peroxide-inducible genes activator	COG0583
at*Ec*_0189	t*Ec*_0177	Lactoylglutathione lyase (EC 4.4.1.5)	COG0346
at*Ec*_0212	t*Ec*_0199	Hydroxyacylglutathione hydrolase (EC 3.1.2.6)	COG0491
at*Ec*_0213	t*Ec*_0200	FIG005121: SAM-dependent methyltransferase (EC 2.1.1.-)	COG0500
**at*Ec*_0417**	**-**	**eKatE, Catalase**	**COG0753**
at*Ec*_0676	t*Ec*_0582	Glutamate--cysteine ligase archaeal (EC 6.3.2.2)	COG2170
at*Ec*_0777	t*Ec*_0682	Ferric uptake regulation protein FUR	COG0735
at*Ec*_0983	t*Ec*_0810	Non-specific DNA-binding protein Dps	COG0783
at*Ec*_1009	t*Ec*_0836	Uncharacterized glutathione S-transferase-like protein	COG0625
at*Ec*_1020	t*Ec*_0847	Glutaredoxin 1	COG0695
at*Ec*_1098	t*Ec*_0925	Hydroxyacylglutathione hydrolase (EC 3.1.2.6)	COG0491
at*Ec*_1102	t*Ec*_0929	Nicotinate phosphoribosyltransferase (EC 2.4.2.11)	COG1488
at*Ec*_1121	t*Ec*_0948	Paraquat-inducible protein A	COG2995
at*Ec*_1122	t*Ec*_0949	Paraquat-inducible protein B	COG3008
at*Ec*_1123	t*Ec*_0950	Paraquat-inducible protein B	COG3009
at*Ec*_1231	t*Ec*_1056	Glutaredoxin 2	COG2999
at*Ec*_1286	t*Ec*_1111	NAD-dependent protein deacetylase of SIR2 family	COG0846
at*Ec*_1499	t*Ec*_1316	Fumarate and nitrate reduction regulatory protein	COG0664
at*Ec*_1624	t*Ec*_1424	NAD-dependent glyceraldehyde-3-phosphate dehydrogenase (EC 1.2.1.12)	COG0057
at*Ec*_1663	t*Ec*_1461	Uncharacterized GST-like protein yncG	COG0625
at*Ec*_1690	t*Ec*_1494	Organic hydroperoxide resistance protein	COG1764
at*Ec*_1936	t*Ec*_1676	Glutathione S-transferase (EC 2.5.1.18)	COG0625
at*Ec*_1947	t*Ec*_1687	Superoxide dismutase [Cu-Zn] precursor (EC 1.15.1.1)	COG2032
at*Ec*_1952	t*Ec*_1692	Lactoylglutathione lyase (EC 4.4.1.5)	COG0346
at*Ec*_1955	t*Ec*_1695	Probable monothiol glutaredoxin GrlA	COG0278
at*Ec*_1957	t*Ec*_1697	Superoxide dismutase [Fe] (EC 1.15.1.1)	COG0605
at*Ec*_2012	t*Ec*_1753	Glutathione peroxidase (EC 1.11.1.9)	COG0386
at*Ec*_2035	t*Ec*_1774	**KatE, Catalase (EC 1.11.1.6)**	COG0753
at*Ec*_2072	t*Ec*_1811	Nicotinamidase (EC 3.5.1.19)	COG1335
at*Ec*_2075	t*Ec*_1814	NAD-dependent glyceraldehyde-3-phosphate dehydrogenase (EC 1.2.1.12)	COG0057
at*Ec*_2129	t*Ec*_1869	Paraquat-inducible protein A	COG2995
at*Ec*_2130	t*Ec*_1870	Paraquat-inducible protein B	COG3008
at*Ec*_2724	t*Ec*_2386	Probable glutathione S-transferase (EC 2.5.1.18), YfcF homolog	COG0625
at*Ec*_2725	t*Ec*_2387	Probable glutathione S-transferase (EC 2.5.1.18), YfcG homolog	COG0625
at*Ec*_3117	t*Ec*_2706	Glutaredoxin-like protein NrdH	COG0695
at*Ec*_3131	t*Ec*_2720	Glutamate--cysteine ligase (EC 6.3.2.2)	COG2918
-	t*Ec*_2780	Organic hydroperoxide resistance transcriptional regulator	ENOG4111Y81
at*Ec*_3470	t*Ec*_3055	Glutathionylspermidine synthase (EC 6.3.1.8)	COG0754
at*Ec*_3471	t*Ec*_3056	Uncharacterized GST-like protein yghU	COG0625
at*Ec*_3474	t*Ec*_3060	Rubredoxin	ENOG4111JT6
at*Ec*_3512	t*Ec*_3106	Similarity with glutathionylspermidine synthase (EC 6.3.1.8), group 1	COG0754
at*Ec*_3577	t*Ec*_3172	Glutathione S-transferase, omega (EC 2.5.1.18)	COG0435
at*Ec*_3682	t*Ec*_3277	Aerobic respiration control sensor protein arcB (EC 2.7.3.-)	COG2198
at*Ec*_3859	t*Ec*_3456	Competence protein F homolog, phosphoribosyltransferase domain	COG1040
at*Ec*_3893	t*Ec*_3490	Gamma-glutamyltranspeptidase (EC 2.3.2.2)	COG0405
at*Ec*_3944	t*Ec*_3541	Glutathione reductase (EC 1.8.1.7)	COG1249
at*Ec*_3985	t*Ec*_3574	Cytochrome c551 peroxidase (EC 1.11.1.5)	COG1858
at*Ec*_4069	t*Ec*_3658	Uncharacterized GST-like protein yibF	COG0625
at*Ec*_4092	t*Ec*_3676	Glutaredoxin 3 (Grx3)	COG0695
at*Ec*_4458	t*Ec*_3984	Manganese superoxide dismutase (EC 1.15.1.1)	COG0605
at*Ec*_4496	t*Ec*_4022	**KatG, Peroxidase (EC 1.11.1.7)**	COG0376
at*Ec*_4514	t*Ec*_4042	OxyR, Hydrogen peroxide-inducible genes activator	ENOG410XNR2
at*Ec*_4604	t*Ec*_4132	Zinc uptake regulation protein ZUR	COG0735
at*Ec*_4620	t*Ec*_4149	Regulatory protein SoxS	ENOG4111IXA
at*Ec*_4621	t*Ec*_4150	Redox-sensitive transcriptional activator SoxR	COG0789
at*Ec*_4750	t*Ec*_4262	Nitrite-sensitive transcriptional repressor NsrR	COG1959
at*Ec*_4758	t*Ec*_4270	Similarity with glutathionylspermidine synthase (EC 6.3.1.8), group 1	COG0754

Genes involved in oxidative stress responses were searched against the whole genes in at*Ec* and t*Ec* strains with a key phrase “oxidative stress” using the CLgenomics software. An orthologous group that each protein belongs to is shown with its eggNOG ID (http://eggnogdb.embl.de/).
